# Hippocampal functional imaging-derived radiomics features for diagnosing cognitively impaired patients with Parkinson’s disease

**DOI:** 10.1186/s12868-025-00938-8

**Published:** 2025-03-28

**Authors:** Wei Zeng, Xiao Liang, Jiali Guo, Weiling Cheng, Zhibiao Yin, Daojun Hong, Fangjun Li, Fuqing Zhou, Xin Fang

**Affiliations:** 1 Jiangxi Provincial Key Laboratory for Precision Pathology and Intelligent Diagnosis, Department of Radiology, the First Affiliated Hospital, Jiangxi Medical College, Nanchang University, 17 Yongwaizheng Street, Nanchang, 330006 People’s Republic of China; 2 Neuroradiology Laboratory, Jiangxi Province Medical Imaging Research Institute, Nanchang, 330006 People’s Republic of China; 3 Department of Neurology, The First Affiliated Hospital, Jiangxi Medical College, Nanchang University, 17 Yongwaizheng Street, Nanchang, 330006 Jiangxi Province People’s Republic of China

**Keywords:** Parkinson’s disease, Cognitively impaired, Hippocampus, Radiomics, Resting-state functional magnetic imaging

## Abstract

**Purpose:**

The aim of this retrospective study was to investigate whether radiomics features derived from hippocampal functional imaging can effectively differentiate cognitively impaired patients from cognitively preserved patients with Parkinson’s disease (PD).

**Methods:**

The study included a total of 89 clinically definite PD patients, comprising 55 who werecognitively impaired and 34 who were cognitively preserved. All participants underwent functional magnetic resonance imaging and clinical assessments. Preprocessed functional data were utilized to derive the amplitude of the low-frequency fluctuations (ALFF), regional homogeneity (ReHo), voxel-mirrored homotopic connectivity (VMHC), and degree centrality (DC). A standardized set of radiomics features was subsequently extracted from the bilateral hippocampi, resulting in a total of 819 features. Following feature selection, the radiomics score (rad-score) and logistic regression (LR) models were trained. Additionally, the Shapley additive explanations (SHAP) algorithm was employed to elucidate and interpret the predictions made by the LR models. Finally, the relationships between the radiomics features derived from hippocampal functional imaging and the scores of the clinical measures were explored to assess their clinical significance.

**Results:**

The rad-score and LR algorithm models constructed using a combination of wavelet features extracted from ReHo and VMHC data exhibited superior classification efficiency. These models demonstrated exceptional accuracy, sensitivity, and specificity in distinguishing cognitively impaired PD patients (CI-PD) from cognitively preserved PD (CP-PD) patients, with values of 0.889, 0.900, and 0.882, respectively. Furthermore, SHAP values indicated that wavelet features derived from ReHo and VMHC were critical for classifying CI-PD patients. Importantly, our findings revealed significant associations between radiomics wavelet features and scores on the Hamilton Anxiety Scale, Non-Motor Symptom Scale, and Montreal Cognitive Assessment in CI-PD patients (*P* < 0.05, with Bonferroni correction).

**Conclusions:**

Our novel rad-score model and LR model, which utilize radiomics features derived from hippocampal functional imaging, have demonstrated their value in diagnosing CI-PDpatients. These models can enhance the accuracy and efficiency of functional MRI diagnosis, suggesting potential clinical applications.

**Clinical trial number:**

Not applicable.

**Supplementary Information:**

The online version contains supplementary material available at 10.1186/s12868-025-00938-8.

## Introduction

Cognitive impairment is one of the most prominent non-motor symptoms of Parkinson’s disease (PD). Individuals diagnosed with PD are six times more likely to experience cognitive impairment than their healthy counterparts are. This condition significantly impacts patients’ social functioning and quality of life [1]. Cognitive symptoms occur in up to 40% of PD patients in the early stages and sometimes appear before the onset of motor symptoms. Owing to the varied clinical presentations of cognitive impairment, its diagnosis remains a challenge in clinical practice.

Recent studies have emphasized the significant role of the hippocampus in the occurrence and progression of cognitive impairment in PD [2]. Both the structure and function of the hippocampus have attracted considerable attention as potential neural underpinnings of cognitive decline [3, 4]. The relationship between atrophy in specific subregions of the hippocampus and cognitive deterioration in PD patients indicates that changes in hippocampal morphology could facilitate the early detection of cognitively impaired PD (CI-PD) patients [5, 6]. Furthermore, functional magnetic resonance imaging (fMRI) studies have demonstrated that alterations in hippocampal activity patterns during cognitive tasks are consistent with cognitive dysfunction [7]. A recent study examining regional variations in gene expression in PD patients with mild cognitive impairment (PD-MCI) revealed that out of 17,216 genes expressed in the hippocampus, 104 genes exhibited differential expression in a PD-MCI mouse model [8]. Collectively, these studies support the potential of the hippocampus as an early predictor of CI-PD.

Resting-state fMRI (rs-fMRI) provides a novel approach for investigating the brain, making it an ideal tool for studying neurodegenerative diseases characterized by brain disconnection syndrome [9]. In the context of CI-PD, rs-fMRI has been employed to explorealterations in intrinsic brain activity, revealing the significant role of the hippocampus in the cognitive decline associated with this condition [7]. Previous studies have demonstrated decreased hippocampal functional connectivity in PD patients [10, 11], as well as reduced degree centrality (DC) and regional homogeneity (ReHo) values linked to cognitive impairment [12–14]. Hippocampal regional dysfunction may serve as an early biomarker for CI-PD, although its recognition remains inconsistent across studies [14]. Some studies have suggested that functional changes in the hippocampus might precede structural alterations [15–17]. However, translating these rs-fMRI findings into clinical practice remains a challenge. Despite advances in neuroimaging, the application of hippocampal functional imaging in diagnosing cognitive impairment in PD remains underexplored. While traditional imaging techniques have provided valuable insights into the structural changes in PD, there is a pressing need for more sophisticated approaches, such as radiomics, to extract and analyse subtle functional abnormalities that may precede clinical symptoms.

Radiomics, a morphological method for image analysis widely utilized in tumor imaging [18], quantitatively detects numerous texture features, allowing for the identification of complex patterns that are not visible to the naked eye. In CI-PD patients, the grayscale distribution of functional images reflects altered blood oxygen level-dependent (BOLD) signals, which are attributed to the regional heterogeneity of neuronal damage within the hippocampus. The higher-order information, including texture features contained in the BOLD signal, may generate a unique imaging characteristic even prior to the manifestation of macroscopic structural changes. Therefore, radiomics can also extract higher-order information that reflects the complexity of neuronal activity in the region of interest. In the context of PD, the application of radiomics to hippocampal functional imaging holds promise for detecting early cognitive decline with greater accuracy than traditional imaging methods.

In this study, we hypothesized that hippocampal radiomics features derived from rs-fMRI could effectively distinguish patients with CI-PD from other PD patients. Furthermore, we posited that rs-fMRI data, including ReHo, amplitude of low-frequency fluctuation (ALFF), DC and voxel-mirrored homotopic connectivity (VMHC), would partially reflect detailed neuropsychological assessments. To achieve this goal, we employed supervised machine learning algorithms to construct a radiomics model based on hippocampal rs-fMRI derivatives aimed at differentiating between PD patients with and without cognitive deficits. Our objective was to explore the potential value of hippocampal functional imaging-derived radiomics features as imaging biomarkers for evaluating cognitive decline in PD patients and to investigate the correlation between these radiomics scores and cognitive function scores.

## Materials and methods

### Participants

A total of 102 PD patients were recruited from the Department of Neurology at the First Affiliated Hospital of Nanchang University between August 2020 and October 2023. All the subjects were right-handed and had no contraindications for MRI. The inclusion criteria were as follows: [1] met the Movement Disorder Society (MDS) clinical diagnostic criteria for PD; [2] were aged above 18 years; and [3] Hoehn–Yahr stage < 5. The exclusion criteria were as follows: [1] severe heart, liver, or kidney diseases; [2] severe mental illness; [3] inability to cooperate with MRI examination and clinical evaluation; and [4] abnormal brain structural changes on conventional MRI. MRI scans and clinical symptom assessments were performed for all the subjects.

This study was conducted in accordance with the Declaration of Helsinki, and ethical approval was obtained from the Ethics Committee of the First Affiliated Hospital of Nanchang University (No. IIT2022124). Written informed consent was obtained from all participants or their legal guardians prior to their involvement in the study.

### Clinical assessment and grouping

Each participant underwent a series of neuropsychological tests designed to assess various aspects of their condition. The severity of motor symptoms was evaluated using the Unified Parkinson’s Disease Rating Scale Part III (UPDRS-III) [19], with higher scores indicating more severe motor impairment. Non-motor symptoms were assessed using the Non-Motor Symptom Scale (NMSS) [20] for PD, where higher scores reflect more severe non-motor impairment. Disease staging was determined by the Hoehn–Yahr stage. Quality of life was assessed using the Parkinson’s Disease Questionnaire-39 (PDQ-39) scale [21], with higher scores indicating a lower quality of life. Depression severity was evaluated using the Hamilton Depression Scale (HAMD-24) [22], which consists of 24 items, whereas anxiety severity was assessed with the Chinese version of the Hamilton Anxiety Scale (HAMA) [23], where higher scores indicate greater severity. Additionally, demographic information, including age, sex, years of education, and disease duration, was collected for each participant.

Cognitive status was evaluated using the Montreal Cognitive Assessment-Beijing Version (MoCA-BJ)(www.mocatest.org) [24, 25]. For participants with less than 12 years of education, 1 point was added to their MoCA score [26, 27]. According to the MDS Working Groupgroup level 1 criteria [28], a MoCA score < 26 was defined as a CI-PD. Those who did not meet these criteria were classified as cognitively preserved PD patients (CP-PD).

### MRI data acquisition and preprocessing

#### MRI protocol

All the subjects underwent MRI scans using a GE 3.0T-SIGNATM Pioneer MRI scanner (GE Healthcare, Milwaukee, WI, USA) equipped with a 24-channel head coil. To effectively minimize head movement, comfortable foam padding was provided to the subjects. Moreover, earplugs were given to them to reduce the noise generated by the scanner. Before the structural MRI data were collected, routine clinical sequences, such as T2-weighted imaging (T2WI), T2 fluid attenuated inversion recovery (T2-FLAIR), MR angiography (MRA), and Diffusion weighted imaging (DWI), were obtained to detect any brain abnormalities.

High-resolution 3D T1-weighted structural images were acquired for each subject using a 3D T1 spoiled gradient echo (SPGR) sequence. The scanning parameters were as follows: repetition time (TR) = 8 ms, echo time (TE) = 3 ms, field of view (FOV) = 220 mm × 220 mm, acquisition matrix = 220 × 220, flip angle (FA) = 12°, slice thickness = 1 mm, slice gap = 0.5, voxel size = 1 × 1 × 1 mm^3^, and a total of 180 slices.

Rs-fMRI data were obtained by the gradient-recalled echo echo-planar imaging (GRE-EPI) sequence. The scanning parameters were as follows: TR = 2000 ms, TE = 25 ms, FA = 90°, FOV = 190 mm × 190 mm, slice thickness = 3.5 mm, matrix = 64 × 64, voxel size = 3.0 × 3.0 × 3.5 mm^3^, interval = 1.2 mm, and a total of 240 time points.

#### Data preprocessing

The rs-fMRI data were initially examined using the MRIcro software package to remove incomplete image extents, artifacts, and significant head motion data. The preprocessing pipeline for the images was subsequently carried out using the Data Processing Assistant for Resting-State fMRI (DPARSF, accessible at http://rfmri.org/DPARSF), which is based on Statistical Parametric Mapping (SPM, available at http://www.fil.ion.ucl.ac.uk/spm/). The main steps implemented for the rs-fMRI data were as follows: [1] the first 10 volumes of each functional time series were removed to account for magnetization equilibrium, and slice timing correction was performed to enhance BOLD signal stability; [2] head motion was corrected by realigning the images to a reference scan; [3] the 3D T1-weighted structural images were spatially registered to the functional images generated in step 2; [4] segmentation of the T1 images into gray matter and white matter and cerebrospinal fluid was performed using the New Segmentation + DARTEL algorithm, followed by normalization of the segmented images to a standard template (Montreal Neurological Institute) using the DARTEL algorithm; [5] spatial normalization of functional images to the Montreal Neurological Institute template and resampling to a voxel size of 3 × 3 × 3 mm^3^; [6] elimination of covariates such as linear drift, white matter signals, and cerebrospinal fluid without employing global signal regression; [7] application of spatial smoothing with a full width at half maximum kernel of 6 mm to the functional images (with the exception of ReHo and DC calculations); and [8] filtering of the data to a frequency range of 0.01–0.1 Hz to reduce respiration and other high-frequency physiological noise.

### Calculation of rs-fMRI derivatives

The following rs-fMRI-based derivatives were calculated [29]: [1] ALFF is calculated as the amplitude of the time series within a certain frequency band (0.01–0.1 Hz), which is the average square root of the power spectral density of the filtered time series [30]. [2] VMHC quantifies functional homotopy by providing a voxel-wise measure of connectivity between hemispheres (with a symmetric template) [31]. [3] ReHo is calculated as the Kendall coefficient of concordance (KCC) among a seed voxel and its 26 neighboring voxels, reflecting the level of spontaneous activity in the seed voxel’s vicinity [32]. [4] DC is a measure of local network connectivity and identifies the most connected nodes by counting the number of direct connections (edges) to all other nodes. Specifically, Pearson correlation analysis was used to calculate the correlation coefficient between the time series of a voxel and the time series of other voxels in the subject’s brain, and then the correlation coefficients of *r* > 0.25 were summed to obtain the degree centrality index of the voxel with weight attributes [33]. After all the images underwent Z-transformation, the ReHo and DC images were spatially smoothed using a Gaussian kernel of 6 mm for subsequent analysis.

### Radiomics feature extraction and selection derived from hippocampal functional imaging

The bilateral hippocampi were segmented using the Anatomical Automatic Labelling (AAL) atlas. Data encapsulation was subsequently carried out using Python 3.7.12 in conjunction with the Pandas library. This was followed by the extraction of a standardized suite of radiomics features using the utilizatiion of the open-source Python package PyRadiomics (version 3.0.1; https://pyradiomics.readthedocs.io/). In total, 819 radiomics features were extracted from the hippocampus. These features were compoesd of 18 first-order features, 73 textural features, and 728 wavelet features for each rs-fMRI derivative (for detailed information, please refer to the Supplementary Material Table S1).

Among the extracted features, many exhibited high redundancy, which complicated classification and increased computational complexity. To identify the optimal features, all the features were first preprocessed through Z-score normalization, with each feature dimension linearly stretched between [0, 1]. Then, forredundancy elimination, we employed the Spearman rank correlation test to evaluate the linear correlation between individual features. If two features exhibited a stronger correlation, the absolute value of their correlation coefficient would be greater. When the Spearman correlation coefficient between any two features was greater than 0.9, we selected one feature for subsequent analysis using a greedy algorithm for feature selection. We subsequently used the least absolute shrinkage and selection operator (LASSO) regression model to identify the optimal radiomics features with non-zero coefficients regarded as valuable predictors [34, 35]. The LASSO regression model is the most commonly used feature selection method [36]. This study employed LassoCV in conjunction with 10-fold cross-validation to automatically select the optimal λ value, thereby optimizing the feature selection process. Specifically, LASSO regression improves the model’s predictive performance and stability by integrating the least squares method—which minimizes prediction error—with L1 regularization, which imposes a penalty on the L1 norm of the regression coefficients. This approach efficiently selects features while adjusting the regularization intensity (α).

### Radiomics score derived from hippocampal functional imaging

The features selected using LASSO were utilized to construct a radiomics score (rad-score), which is a quantitative index derived from radiomics features. For each patient, this score was calculated by a linear combination of the selected features, weighted by their respective coefficients. For more detailed information, please refer to the formula provided in Supplementary Material item 2.

Receiver operating characteristic (ROC) curve analysis and Youden’s index were employed to determine the optimal threshold of the radscore for differentiating between CI-PD patients and CP-PD patients. This process was confined to the training cohort framework, meaning that the rad-score threshold was generated entirely from the data of the training cohort. The area under the ROC curve (AUC) was used to evaluate the performance. The sensitivity and specificity, along with their corresponding 95% confidence intervals (CIs), were computed. Moreover, the rad-score was generated for patients in the internal validation cohort to validate the model. Specifically, the selected threshold was applied to the validation cohort to obtain metrics such as sensitivity, specificity, accuracy, and other relevant metrics.

### Machine learning model constructed with hippocampal radiomics features

After feature selection, the discriminative ability of radiomics features in distinguishing between CP-PD patients and CI-PD patients was evaluated. Machine learning classification models were constructed using Python 3.7.12 and the Scikit-learn library. The patients were divided into training and validation cohorts at a 7:3 ratio. To address the unequal distribution of the two classes, we used a balanced sampling technique, namely, the synthetic minority oversampling technique (SMOTE). The features selected from the training cohort were used as input vectors for logistic regression (LR) to train the classification model, which was subsequently validated in the validation cohort.

The diagnostic performance of the radiomics models was evaluated using the area under the curve (AUC) of the receiver operating characteristic (ROC) curve in both the training and validation cohorts, alongside quantitative indicators such as accuracy (ACC), sensitivity (SEN), and specificity (SPE). Decision curve analysis (DCA) was conducted to evaluate the clinical utility of the prediction models in the validation cohort.

Understanding and determining the significance and impact of each feature in predictive models presents a considerable challenge. The Shapley additive explanations (SHAP) analysis serves as a valuable tool for evaluating feature importance. Unlike black-box models, such as deep neural networks, SHAP analysis elucidates the relationship between inputs and outputs through a series of easily interpretable if-then rules. This methodology provides a globally interpretable framework that employs optimal Shapley values to quantify the contribution of each feature within the model. Furthermore, SHAP analysis delivers interpretable predictions for machine learning classifiers, effectively addressing the limitations of traditional models that often lack clear feature directionality. In the context of diagnosing cognitively impaired patients with Parkinson’s disease, SHAP-based feature selection offers a more interpretable approach for assessing the importance of hippocampal functional image-derived radiomics features.

### Different combinations of radiomics features for modelling

Each radiomics feature of the derivative/connectivity measurements captures a distinct aspect of the information conveyed by rs-fMRI and may play a unique role in differentiating between patients with PD-CP and those with PD-CI. In addition to the previously mentioned combination of features (ALFF + DC + ReHo + VMHC), 14 other combinations were examined, such as ALFF, DC, ReHo, VMHC, ALFF + ReHo, ALFF + DC, ALFF + VMHC, DC + ReHo, DC + VMHC, ReHo + VMHC, ALFF + DC + ReHo, ALFF + DC + VMHC, ALFF + ReHo + VMHC, and DC + ReHo + VMHC. The analysis methodology for these combinations was similar to that used for ALFF + DC + ReHo + VMHC. A comparison was made between the results obtained using 15 combinations, with a focus on the classification outcomes.

### Statistical analysis

Statistical analyses were performed using GraphPad Prism 9 (GraphPad, San Diego, CA, USA) and IBM SPSS Statistics (Version 21.0, USA). Chi-square tests were used to compare categorical variables, whereas 2-sample t tests or Mann–Whitney U tests were used to compare quantitative variables to evaluate the differences in the clinical characteristics of patients. All tests were two-sided, with statistical significance set at *P* < 0.05.

To explore the relationships between radiomics features and clinical measures, a partial correlation analysis was performed between the selected features and clinical scale scores (MoCA scores, UPDRS-III scores, NMSS scores, PDQ-39 scores, HAMA scores, HAMD scores, BMI, years of education, and disease duration) in patients with CI-PD and CP-PD, with age considered a covariate (P × *n* < 0.05, Bonferroni correction).

To investigate the significance of the performance of the rad-score model and LR model in different combinations, the differences between various AUCs were compared using the DeLong test.

## Results

### Demographic and clinical data profiling

Among the 102 patients initially recruited, a total of 13 patients were excluded from the study. This exclusion consisted of four individuals due to incomplete MRI examinations, four for not completing the clinical scales, and five for excessive head movement. The final analysis included 89 PD patients, comprising 34 CP-PD patients and 55 CI-PD patients. The patients were randomly divided into a training cohort and a validation cohort at a 7:3 ratio. The data of the validation cohort were used to evaluate the performance of the models. The demographic and clinical data are presented in Table 1. Notably, CP-PD patients had a higher education level than CI-PD patients did. However, there were no significant differences between the two groups in terms of age, sex, UPDRS-III score, NMSS score, PDQ-39 score, HAMA score, HAMD score, BMI score, or disease duration.

**Table 1:** Demographic and clinical characteristics of CI-PD and CP-PD patients

	PD-CI (*N* = 55)	PD-CP (*N* = 34)	*P* value
Gender (M/F)	29/26	19/15	0.772^a^
Age, years (mean ± SD)	61.56 ± 7.61	56.68 ± 10.39	0.039^b^
BMI (mean ± SD)	22.02 ± 2.68	22.92 ± 1.98	0.193 ^b^
Education years (median (IQR))	5.00 (4.00)	9.00 (5.00)	< 0.001^c^
Disease duration, years (median (IQR))	3.50 (3.00)	2.00(2.60)	0.091 ^c^
Montreal Cognitive Assessment (median (IQR))	18.00 (7.00)	27.00 (1.00)	< 0.001^c^
HAMA (median (IQR))	10.00 (11.00)	9.00 (11.00)	0.273 ^c^
HAMD (median (IQR))	10.00 (8.00)	6.00 (8.00)	0.130 ^c^
UPDRS-III (mean ± SD)	41.05 ± 13.38	34.12 ± 13.33	0.475^b^
NMSS (mean ± SD)	53.13 ± 33.94	47.35 ± 29.32	0.197 ^b^
PDQ-39 (mean ± SD)	46.75 ± 20.45	38.41 ± 23.11	0.188 ^b^

Note: CP-PD, cognitively preserved Parkinson’s disease; CI-PD, cognitively impaired Parkinson’s disease; HAMD, Hamilton Depression Scale; HAMA, Hamilton Anxiety Scale; NMSS, Non-Motor Symptom Scale; UPDRS-III, Unified Parkinson’s Disease Rating Scale Part III; PDQ-39, Parkinson’s Disease Questionnaire-39; F, female; M, male; SD, standard deviation; IQR, interquartile range; ^a^, chi-square test; ^b^, 2-sample t test; ^c^, Mann‒Whitney U test

### Hippocampal functional radiomics features selection

**Fig. 1:**
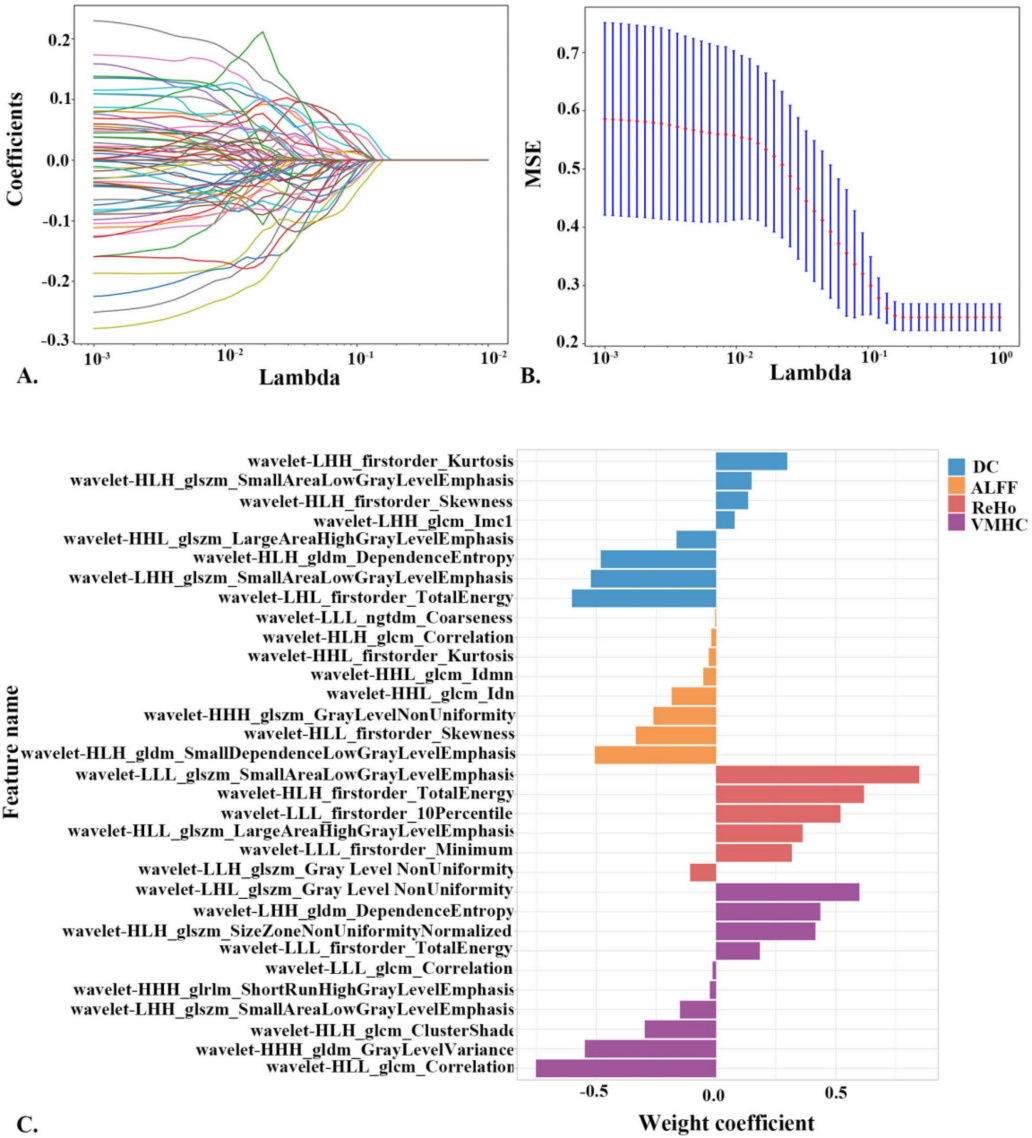
Radiomics features dimension reduction. (**A**) LASSO coefficient profiles of the features. Different color lines show the corresponding coefficient of each feature. (**B**) Tuning parameter (lambda) selection in the LASSO model. (**C**) Selected feature weight coefficients, Note: LASSO, least absolute shrinkage and selection operator; MSE, mean square error; ALFF, amplitude of low-frequency fluctuations; ReHo, regional homogeneity; DC, degree centrality; VMHC, voxel-mirrored homotopic connectivity

For 3276 (819 × 4)features, following feature screening based on a Spearman correlation coefficient threshold of > 0.9, a total of 776 features (24%) were retained. This included 170 ALFF features (22%), 187 DC features (24%), 184 ReHo features (24%), and 235 VMHC features (30%). Subsequently, LASSO regression was applied for feature selection, identifying non-zero coefficients as valuable predictors within each feature group. Ultimately, 8 ALFF features, 6 ReHo features, 8 DC features, and 10 VMHC features were retained for distinguishing between patients with CI-PD and those with CP-PD (refer to Fig. 1; Table 2). These radiomics features encompassed 8 features from ALFF, 6 from ReHo, 10 from VMHC, and 8 from DC. Notably, all the selected features were wavelet features, with particular significance attributed to the VMHC wavelet features in distinguishing between CI-PD and CP-PD patients. Additional results for various feature combinations using the same feature selection strategy were presented in Supplementary Material Tables S2–15. Similarly, the results for features selected from the GMV are presented in Supplementary Material Table S16.

**Table 2:** The selected radiomics features of ALFF, DC, ReHo and VMHC

	Feature	Weight coefficients
ALFF derived radiomics features		
1	wavelet-HHH_glszm_GrayLevelNonUniformity	-0.0262
2	wavelet-HHL_firstorder_Kurtosis	-0.0030
3	wavelet-HHL_glcm_Idmn	-0.0053
4	wavelet-HHL_glcm_Idn	-0.0185
5	wavelet-HLH_glcm_Correlation	-0.0020
6	wavelet-HLH_gldm_SmallDependenceLowGrayLevelEmphasis	-0.0507
7	wavelet-HLL_firstorder_Skewness	-0.0335
8	wavelet-LLL_ngtdm_Coarseness	-0.0004
DC derived radiomics features		
1	wavelet-HHL_glszm_LargeAreaHighGrayLevelEmphasis	-0.0165
2	wavelet-HLH_firstorder_Skewness	0.0134
3	wavelet-HLH_gldm_DependenceEntropy	-0.0482
4	wavelet-HLH_glszm_SmallAreaLowGrayLevelEmphasis	0.0148
5	wavelet-LHH_firstorder_Kurtosis	0.0297
6	wavelet-LHH_glcm_Imc1	0.0077
7	wavelet-LHH_glszm_SmallAreaLowGrayLevelEmphasis	-0.0523
8	wavelet-LHL_firstorder_TotalEnergy	-0.0602
ReHo derived radiomics features		
1	wavelet-HLH_firstorder_TotalEnergy	0.0618
2	wavelet-HLL_glszm_LargeAreaHighGrayLevelEmphasis	0.0361
3	wavelet-LLH_glszm_Gray Level NonUniformity	-0.0109
4	wavelet-LLL_firstorder_10Percentile	0.0520
5	wavelet-LLL_firstorder_Minimum	0.0317
6	wavelet-LLL_glszm_SmallAreaLowGrayLevelEmphasis	0.0849
VMHC derived radiomics features		
1	wavelet-HHH_gldm_GrayLevelVariance	-0.0548
2	wavelet-HHH_glrlm_ShortRunHighGrayLevelEmphasis	-0.0026
3	wavelet-HLH_glcm_ClusterShade	-0.0298
4	wavelet-HLH_glszm_SizeZoneNonUniformityNormalized	0.0415
5	wavelet-HLL_glcm_Correlation	-0.0753
6	wavelet-LHH_gldm_DependenceEntropy	0.0436
7	wavelet-LHH_glszm_SmallAreaLowGrayLevelEmphasis	-0.0151
8	wavelet-LHL_glszm_Gray Level NonUniformity	0.0599
9	wavelet-LLL_firstorder_TotalEnergy	0.0183
10	wavelet-LLL_glcm_Correlation	-0.0015

Note: ALFF, amplitude of low-frequency fluctuations; ReHo, regional homogeneity; DC, degree centrality; VMHC, voxel-mirrored homotopic connectivity; glcm, gray-level co-occurrence matrix; glrlm, gray-level run-length matrix; glszm, gray-level size zone matrix; ngtdm, neighborhood gray-tone difference matrix; gldm, gray-level dependence matrix

### Classification performance of the hippocampal functional radiomics score

**Fig. 2:**
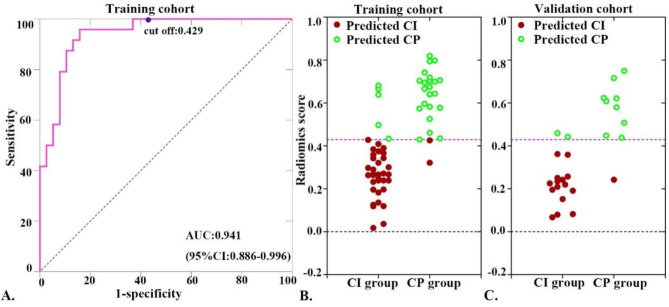
The cut-off value obtained from receiver operating characteristic (ROC) curves of the rad-score model (**A**), which was constructed using two hippocampal functional derivative (ReHo- and VMHC)-derived radiomics features. This cut-off value was highly sensitive in discriminating between patients with CI-PD and those with CP-PD in both the training cohort (**B**) and the validation cohort (**C**)

The classification performance of the hippocampal functional rad-score, derived from selected radiomics features, including 15 combinations, is presented in Supplementary Material Table S17. Notably, the combination-based rad-score was constructed using two functional derivatives (ReHo and VMHC), resulting in superior classification performance, with an AUC of 0.941. Furthermore, all the evaluation metrics, including the ACC, SPE, and SEN, exceeded 0.850. The optimal cut-off value for distinguishing between CI-PD patients and CP-PD patients in the training cohort was determined to be 0.429 on the basis of Youden’s index derived from the ROC curves (Fig. 2A). This threshold not only sensitively discriminated between patients with CI-PD and CP-PD in the training cohort (Fig. 2B) but also effectively distinguished between these two groups, achieving high values of ACC (0.889), SPE (0.900), and SEN (0.882) (Fig. 2C and Table S17).

### Classification performance of the machine learning model

**Fig. 3:**
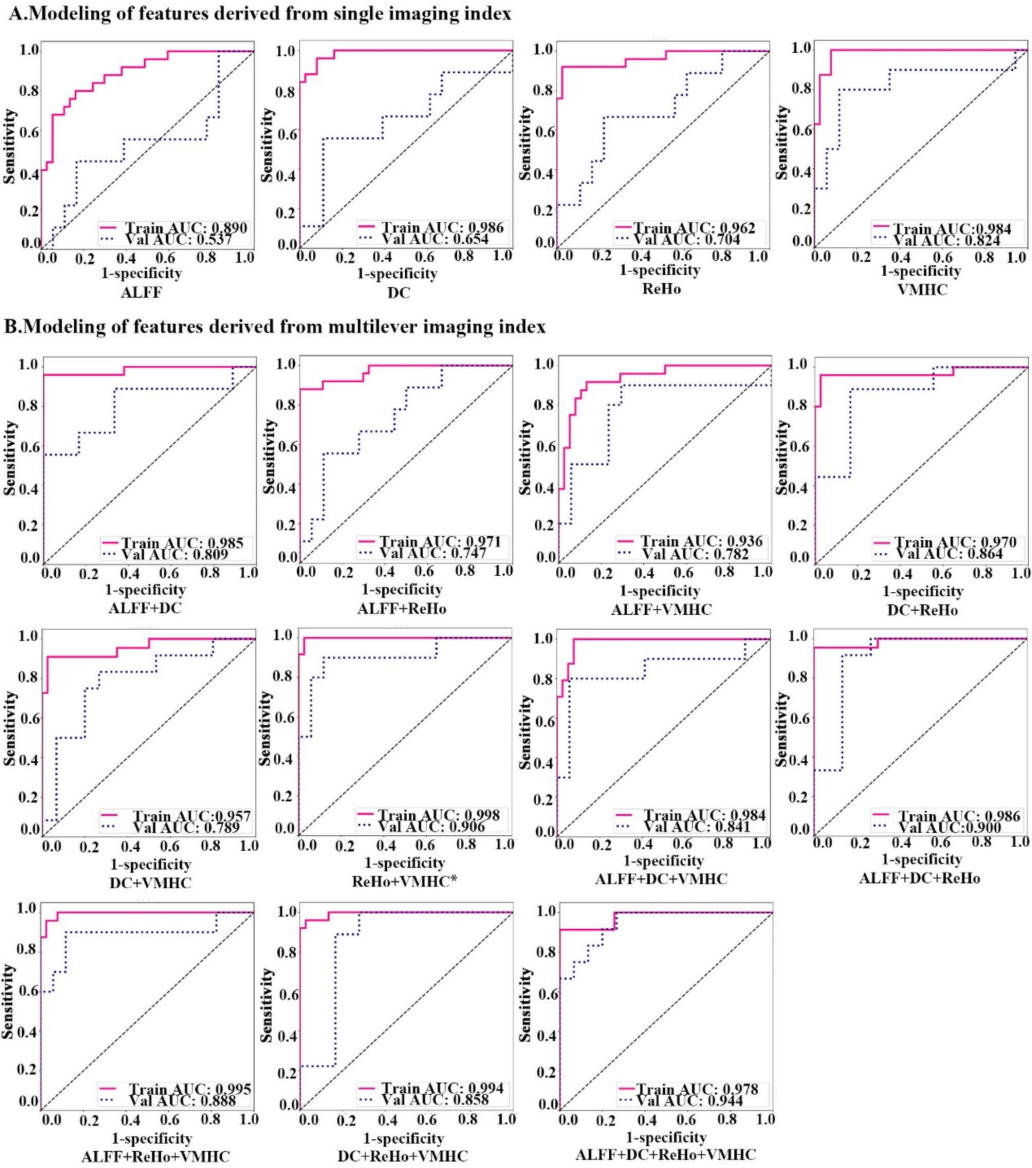
Selected hippocampal functional radiomics features were used to construct LR models with different feature combinations. * the optimal combination. Note: AUC, area under curve; Val, validation, LR, logistic regression; ALFF, amplitude of low-frequency fluctuations; ReHo, regional homogeneity; DC, degree centrality; VMHC, voxel-mirrored homotopic connectivity

The LR models were trained using 32 selected radiomics features derived from ALFF, ReHo, VMHC, and DC, with 15 combinations shown in Fig. 3; Table 3. Compared with those derived from a single functional derivative, the radiomics features obtained from combined functional derivatives demonstrated superior performance. Notably, the model constructed using radiomics features from two functional derivatives (ReHo and VMHC) achieved an AUC value of 0.906 for the validation dataset, with the ACC, SEN, and SPE values all exceeding 0.850. Furthermore, the results of the DCA demonstrated that the LR model constructed with the combined radiomics features of ReHo and VMHC has potential for clinical application (see Supplementary Material Figure S1). The high net benefit and stable change over a wide range of threshold probabilities indicate that the model has good generalizability and consistent performance. Additionally, the classification performance of the models constructed using radiomics features from GMV is presented in Supplementary Material Table S18.

This study compared the diagnostic performance of the rad-score models and LR models, as well as various combinations within these groups. The DeLong test was utilized to analyse the performance of the models, with the results presented in Supplementary Material Tables S19–21. When comparing the combination of ReHo + VMHC within the rad-score models, although the AUCs for DC, DC + ReHo, and ALFF + ReHo + VMHC were greater, their differences lacked statistical significance, as indicated by the DeLong test results (refer to Supplementary Material Table S19). In the LR models, the combination of ReHo + VMHC showed that the addition of ALFF or DC did not significantly enhance model performance. In contrast, incorporating both ALFF and DC yielded a higher AUC of 0.944, but this difference was not statistically significant according to the results of the DeLong test (see Supplementary Material Table S20). For the combination of ReHo + VMHC, the AUCs of the rad-score models were not significantly different from those of the LR models (details in Supplementary Material Table S21).

**Fig. 4:**
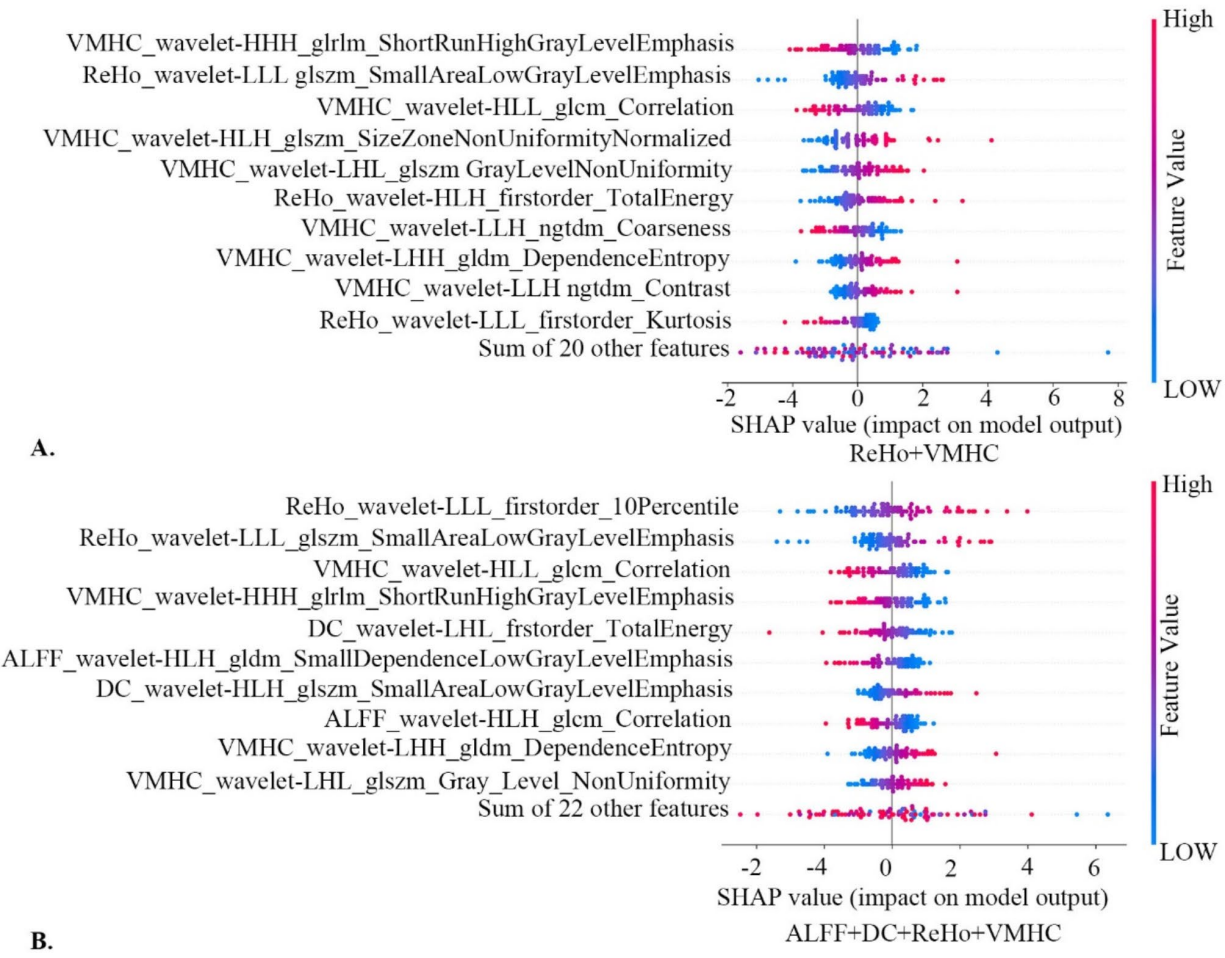
Relative importance of features based on SHAP for the LR classification model. Note: SHAP, Shapley’s additive explanations; LR, logistic regression. ALFF, amplitude of low-frequency fluctuations; ReHo, regional homogeneity; DC, degree centrality; VMHC, voxel-mirrored homotopic connectivity.

SHAP analysis of the ReHo + VMHC model revealed that the features VMHC_wavelet-LLL_GLscrcrshortareal_owgrayLevelEmphasis, ReHo_wavelet-LLL_GLscrcrsmallarealowgrayLevelEmphasis, and VMHC_wavelet-HLL_glcm_Correlation are significant indicators for diagnosing CI-PD (see Fig. 4A). Furthermore, similar results were observed in the SHAP analysis of the combined ALFF + ReHo + DC + VMHC model (see Fig. 4B). The SHAP summary plot for the remaining 13 models is available in the Supplementary Materials, Figure S2-S4.

**Table 3:** The LR model was constructed with different hippocampal radiomics features in the training and validation cohorts to discriminate between CI-PD patients and CP-PD patients

LR models	Task	AUC(95%CI)	ACC	SEN	SPE
Modelling of hippocampal radiomics features derived from single imaging metrics					
ALFF	Training cohort	0.890(0.809–0.970)	0.823	0.800	0.838
	Validation cohort	0.537(0.276–0.789)	0.704	0.444	0.882
DC	Training cohort	0.986(0.967-1.000)	0.935	0.960	0.919
	Validation cohort	0.654(0.404–0.905)	0.778	0.556	0.889
ReHo	Training cohort	0.962(0.913-1.000)	0.952	0.920	0.973
	Validation cohort	0.704(0.484–0.923)	0.741	0.667	0.778
VMHC	Training cohort	0.984(0.960-1.000)	0.952	1.000	0.921
	Validation cohort	0.824(0.672-1.000)	0.852	0.800	0.882
Modelling of hippocampal radiomics features derived from two imaging metrics					
ALFF + DC	Training cohort	0.985(0.955-1.000)	0.984	0.960	1.000
	Validation cohort	0.809(0.602-1.000)	0.852	0.556	1.000
ALFF + ReHo	Training cohort	0.971(0.934-1.000)	0.952	0.880	1.000
	Validation	0.747(0.550–0.944)	0.778	0.556	0.889
ALFF + VMHC	Training cohort	0.936(0.876–0.996)	0.887	0.917	0.868
	Validation cohort	0.782(0.572–0.993)	0.778	0.900	0.706
DC + ReHo	Training cohort	0.970(0.918-1.000)	0.968	0.960	0.973
	Validation cohort	0.864(0.716-1.000)	0.852	0.889	0.833
DC + VMHC	Training cohort	0.957(0.901-1.000)	0.952	0.909	0.975
	Validation cohort	0.789(0.606–0.972)	0.778	0833	0.733
ReHo + VMHC*	Training cohort	0.998(0.993-1.000)	0.984	1.000	0.974
	Validation cohort	0.906(0.770-1.000)	0.889	0.900	0.882
Modelling of hippocampal radiomics features derived from three imaging metrics					
ALFF + DC + ReHo	Training cohort	0.986(0.959-1.000)	0.984	0.955	1.000
	Validation cohort	0.900(0.774-1.000)	0.889	0.917	0.867
ALFF + DC + VMHC	Training cohort	0.984(0.961-1.000)	0.952	1.000	0.921
	Validation cohort	0.841(0.652-1.000)	0.889	0.800	0.941
ALFF + ReHo + VMHC	Training cohort	0.995(0.985-1.000)	0.968	0.958	0.974
	Validation cohort	0.888(0.723-1.000)	0.889	0.900	0.882
DC + ReHo + VMHC	Training cohort	0.994(0.981-1.000)	0.968	0.960	0.973
	Validation cohort	0.858(0.710-1.000)	0.852	0.889	0.833
Modelling of hippocampal radiomics features derived from four imaging metrics					
ALFF + DC + ReHo + VMHC	Training cohort	0.978(0.945-1.000)	0.968	0.913	1.000
	Validation cohort	0.944(0.869-1.000)	0.852	1.000	0.733

Note: AUC, area under the curve; ACC, accuracy; SEN, sensitivity; SPE, specificity; LR, logistic regression; ALFF, amplitude of low-frequency fluctuations; ReHo, regional homogeneity; DC, degree centrality; VMHC, voxel-mirrored homotopic connectivity. * the optimal combination

### Correlation analysis

**Fig. 5:**
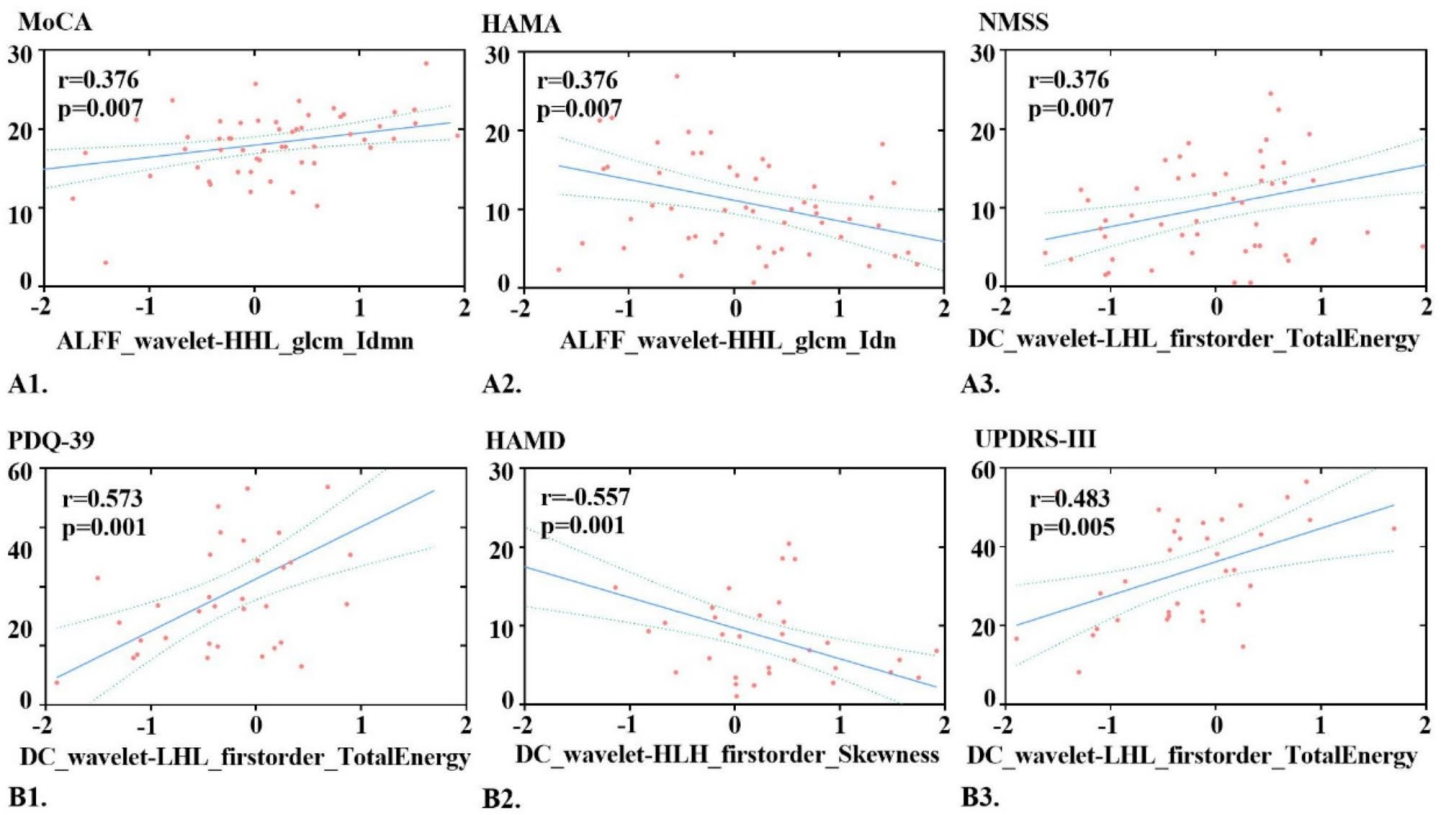
Partial correlation analysis between clinical scales and radiomics features in the CI-PD group (**A1**–**A3**) and CP-PD group (**B1**–**B3**). Note: CP-PD, cognitively preserved Parkinson’s disease; CI-PD, cognitively impaired Parkinson’s disease; HAMD, Hamilton Depression Scale; HAMA, Hamilton Anxiety Scale; NMSS, Non-Motor Symptom Scale; PDQ-39, Parkinson’s Disease Questionnaire-39; UPDRS-III, Unified Parkinson’s Disease Rating Scale Part III; MoCA, Montreal Cognitive Assessment; ALFF, amplitude of low-frequency fluctuations; DC, degree centrality

A study conducted on the CI-PD and CP-PD groups revealed significant correlations between various brain imaging features and clinical assessment scores (see Fig. 5, Supplementary Material Table S22-24).

In CI-PD patients, a positive correlation was detected between ALFF-derived wavelet features (wavelet-HHL_glcm_Idmn) and MoCA scores (*r* = 0.376, *p* = 0.007 × 7 < 0.05, with Bonferroni correction); a negative correlation was detected between ALFF-derived wavelet features (wavelet-HHL_glcm_Idn) and HAMA scores (*r* = -0.376, *p* = 0.006 × 7 < 0.05, with Bonferroni correction); and a positive correlation was detected between DC-derived wavelet features (wavelet-LHL_firstorder_TotalEnergy) and NMSS scores (*r* = 0.410, *p* = 0.003 × 7 < 0.05, with Bonferroni correction).

In CP-PD patients, positive correlations were identified between DC-derived wavelet features (wavelet-LHL_firstorder_TotalEnergy) and PDQ-39 scores (*r* = 0.579, *p* = 0.001 × 7 < 0.05, with Bonferroni correction), as well as between DC-derived wavelet features (wavelet-LHL_firstorder_TotalEnergy) and UPDRS-III scores (*r* = 0.483, *p* = 0.005 × 7 < 0.05, with Bonferroni correction). Conversely, a negative correlation was found between DC-derived wavelet features (wavelet-HLH_firstorder_Skewness) and HAMD scores (*r* = -0.557, *p* = 0.001 × 7 < 0.05, with Bonferroni correction).

## Discussion

This study emphasized the predictive performance within a radiomics framework. The findings of our study demonstrate that the nove*l* rad-score and LR model, which is based on radiomics features derived from hippocampal functional imaging, may serve as a valuable tool for clinical diagnosis by effectively distinguishing between CI-PD patients and CP-PD patients. The combination of wavelet features extracted from ReHo and VMHC demonstrated superior performance compared with other combined features in both the rad-score classification and the LR models. Our findings highlight the clinical relevance of hippocampal functional radiomics features. To the best of our knowledge, this is the first study to investigate hippocampal functional imaging-derived radiomics in a PD cohort.

### Rad-score of hippocampal functional radiomics features

In this study, we present a novel rad-score model that integrats radiomics data derived from hippocampal functional imaging to effectively differentiate between CI-PD patients and CP-PD patients. For 9 out of 15 combinations of radiomics features derived from hippocampal rs-fMRI, both the training and validation cohorts achieved AUCs exceeding 0.90. The diagnostic ability of the hippocampal rad-score for identifying CI-PD was significantly superior to that reported in previous studies in which rs-fMRI-derived imaging techniques were used [37–39]. Notably, the combination-based rad-score was constructed using two functional derivatives (ReHo and VMHC), achieving an impressive AUC of 0.941, with all the metrics, including the ACC, SPE, and SEN, surpassing 0.850, thereby demonstrating outstanding classification performance.

The results of the DeLong test further substantiated our hypothesis that augmenting functional derivatives does not increase the differentiation efficiency of the rad-score. Combining radiomics derived from ReHo and VMHC appeared to be a favourable choice, as it achieved improved performance with fewer indices. This combination was particularly advantageous because of the greater heterogeneity of hippocampal changes observed in CI-PD patients than in CP-PD patients [8, 40, 41]. Moreover, hippocampal ReHo exhibited better complementarity in assessing alterations in homotopic connectivity. The changes observed in hippocampal ALFF and DC may align with those observed in ReHo. However, confirmation of this assertion will require further research.

### Machine learning model constructed with hippocampal functional radiomics features

Our study presented an LR model that utilized functional radiomics features derived from various rs-fMRI metrics, including ReHo, ALFF, VMHC, and DC, specifically in the hippocampus, to classify patients with CI-PD and those with CP-PD. The incorporation of these multilevel rs-fMRI characteristics has been shown to enhance diagnostic accuracy.

Additionally, we constructed an optimal LR model by incorporating features derived from both VMHC and ReHo. Notably, VMHC plays a crucial role, as cognitive and motor functions are known to be associated with brain lateralization. In complex tasks, both hemispheres are typically involved; however, one hemisphere usually dominates the function [42, 43]. PD is a classic example of a lateralized disease [44], and impaired interhemispheric communication has been observed in previous studies [45–47]. Cognitive activities rely on coordination between various brain regions, particularly between the two hemispheres. Abnormal VMHC values in the bilateral hippocampus suggest disrupted information exchange within these regions, potentially leading to cognitive impairment in patients with PD.

To address the ‘black-box’ nature of machine learning models, we employed the SHAP method to interpret the models and identify feature importance. SHAP assigns numerical values that indicate the magnitude and direction of each feature’s contribution to the model’s predictions. The results from the SHAP analysis further emphasized the importance of hippocampal VMHC features in the identification of CI-PD.

Although our LR model did not exhibit significant improvement in performance compared with the rad-score, as determined by statistical analysis using the DeLong test, the rad-score proved to be a cost-effective tool for diagnosing CI-PD in clinical settings. Consequently, the rad-score emerged as a straightforward, practical, and economical method for classifying both CI-PD and CP-PD.

#### Hippocampal functional radiomics features in relation to clinical characteristics

Previous studies have consistently demonstrated a strong inverse correlation between quality of life (measured by the PDQ-39) and performance on various cognitive tests, including the MMSE, MoCA, and Fascat [48]. These findings indicate that individuals with greater cognitive ability tend to experience better quality of life in PD. The findings of this research align with this trend, as radiomics features indicative of cognitive impairment in Parkinson’s disease patients are significantly correlated with PDQ-39 scores. A four-year follow-up study on a PD cohort demonstrated that cognitive decline was linked to depressive symptoms but not to anxiety [49]. However, our current study revealed a significant correlation between HAMA scores and radiomics features, which was observed only in the CI-PD group. This finding may be attributed to the enhanced capacity of radiomics analysis to uncover potential links between anxiety symptoms and cognitive impairment in PD patients. Additionally, the hippocampus may play a crucial role, either directly or indirectly, in the manifestation of cognitive deficits and anxiety.

In addition to depression, other risk factors for cognitive dysfunction in PD patients have been identified, including lower scores on motor symptom tests, rigidity, and postural instability [1]. A previous study suggested that postural instability, as evaluated by the UPDRS-III score, may serve as a potential indicator of the risk for developing cognitive impairment in newly diagnosed PD patients [50]. Motor symptoms have been widely linked to cognitive deficits [51]. Similarly, the present study revealed a positive correlation between UPDRS-III scores and radiomics features in CI-PD patients, indicating that higher UPDRS-III scores are associated with worse cognitive function.

### Limitation

This study has several limitations. First, this was a single-center cross-sectional study, and the predictive model was not externally validated. Therefore, future research should incorporate external validation to confirm the model’s validity. Additionally, the number of features remained relatively high compared with the sample size, which raised the possibility of overfitting. Thus, increasing the sample size and reducing the data dimensionality are necessary. The use of multicenter data would increase the generalizability and stability of our results. Second, various subtypes of CI-PD exist, and this study did not consider the classification of these subtypes. Third, the rad-score and LR models, which were constructed using four functional images in this study, demonstrated a comparable ability to distinguish CI-PD and CP-PD patients compared with the response model based on structural MRI-GMV (see Tables S16, S18). Future studies should include additional functional indices to determine whether they can enhance classification performance.

## Conclusion

The rad-score and LR models, which are based on radiomic features derived from hippocampal functional imaging, serve as valuable tools for diagnosing patients with cognitive impairment related to Parkinson’s disease (CI-PD). These models enhanced the diagnostic accuracy and efficiency of functional MRI, facilitating earlier and more personalized interventions, which ultimately improved patient outcomes.

## Electronic supplementary material

Below is the link to the electronic supplementary material.

Supplementary Material 1

## Data Availability

No datasets were generated or analysed during the current study.
