# Supplementary material for: Hippocampal functional imaging-derived radiomics features for diagnosing cognitively impaired patients with Parkinson’s disease
Source: BMC Neurosci. 2025 Mar 28;26:27. doi: 10.1186/s12868-025-00938-8 (PMC11954276; doi:10.1186/s12868-025-00938-8)
Supplement: Supplementary file 1 — Supplementary Material 1 [file 12868_2025_938_MOESM1_ESM.docx]

**Supplementary Material**

1. **3D-T1 data prepocessing and hippocampal radiomics feature**

Use toolbox of CAT 12 in the software of Statistic Parameter Mapping 12 (SPM 12) (http://www.fil.ion.ucl.ac.uk/spm/software/spm12) to preprocess the T1-weighted MRI data. The main steps of preprocessing are as follows: (1) Format transformation: individual T1-weighted images of "DCM" format data was transformed into "nii" format data by using MRIcron/dicom2nii. (2) Spatial normalisation: The Montreal Neurological Institute (MNI) is a standard brain spatial coordinate system established by theMontreal Neurological Institute of Canada based on a large number of magnetic resonance images of normal subjects. The head of each subject were normalized to the MNI using affine and nonlinear regression algorithms. (3) Bias field correction: Since the MR bias field affects the accuracy of voxel values, a bias field correction will be applied to the image. (4) Segmentation: Tissue segmentation based on tissue probability map and the brain tissues was segmented into gray matter, white matter and cerebrospinal fluid. (5) Modulation: In order to compare volume differences, the segmentation results should be modulated to maintain the invariance of the individual tissue volume.

The radiomics features extracted from the GMV map of each ROI underwent a similar processing method as the rs-fMRI metrics, including feature extraction and selection. Furthermore, we developed GMV-derived radiomics analyses using similar analytical strategies as described above. Additionally, Rad-score and LR algorithm models were also constructed, with results provided in Table S16-17.

1. **The calculation formula of Rad-score**

Radiomics score=0.3855-(0.0262×wavelet-HHH_glszm_GrayLevelNonUniformity)-(0.003×wavelet-HHL_firstorder_Kurtosis)-(0.0053×wavelet-HHL_glcm_Idmn)-(0.0185×wavelet-HHL_glcm_Idn)-(0.0020×wavelet-HLH_glcm_Correlation)- (0.0507×wavelet-HLH_gldm_SmallDependenceLowGrayLevelEmphasis)-(0.0335×wavelet-HLL_firstorder_Skewness)-(0.0004×wavelet-LLL_ngtdm_Coarseness)-(0.0165×wavelet-HHL_glszm_LargeAreaHighGrayLevelEmphasis)+(0.0134×wavelet-HLH_firstorder_Skewness)-(0.0482×wavelet-HLH_gldm_DependenceEntropy)+(0.0148×wavelet-HLH_glszm_SmallAreaLowGrayLevelEmphasis)+(0.0297×wavelet-LHH_firstorder_Kurtosis)+(0.0077×wavelet-LHH_glcm_Imc1)-(0.0523×wavelet-LHH_glszm_SmallAreaLowGrayLevelEmphasis)-(0.0602×wavelet-LHL_firstorder_TotalEnergy)+(0.0618×wavelet-HLH_firstorder_TotalEnergy)+(0.0361×wavelet-HLL_glszm_LargeAreaHighGrayLevelEmphasis)-(0.0108×wavelet-LLH_glszm_Gray Level NonUniformity)+(0.0520×wavelet-LLL_firstorder_10Percentile)+(0.0317×wavelet-LLL_firstorder_Minimum)+(0.0850×wavelet-LLL_glszm_SmallAreaLowGrayLevelEmphasis)-(0.0548×wavelet-HHH_gldm_GrayLevelVariance)-(0.0026×wavelet-HHH_glrlm_ShortRunHighGrayLevelEmphasis)-(0.0298×wavelet-HLH_glcm_ClusterShade)+(0.0415×wavelet-HLH_glszm_SizeZoneNonUniformityNormalized)-(0.0753×wavelet-HLL_glcm_Correlation)+(0.0436×wavelet-LHH_gldm_DependenceEntropy)-(0.0151×wavelet-LHH_glszm_SmallAreaLowGrayLevelEmphasis)+(0.0599×wavelet-LHL_glszm_Gray Level NonUniformity)+(0.0183×wavelet-LLL_firstorder_TotalEnergy)-(0.0015×wavelet-LLL_glcm_Correlation),where glcm is the grey-level co-occurrence matrix, glszm is the grey-level size zone matrix, and gldm is the grey-level dependence matrix. Our Radscore comprised 32 features, all of them were wavelet features. Texture features indicate the quantification of heterogeneity and ROI texture and reflect the interaction between neighboring pixels. Wavelet transform uses a wavelet function to decompose the original image and obtain wavelet-based features.

Table S1. Detailed information of the radiomics features.

| category | | NF | Features |
| --- | --- | --- | --- |
| First order features | | 18 | Energy, Total Energy, Entropy, Minimum, 10th percentile, 90th percentile, Maximum, Mean, Median, Interquartile Range, Range, Mean Absolute Deviation, Robust Mean Absolute Deviation, Root Mean Squared, Skewness, Kurtosis, Variance, Uniformity |
| Texture features | GLCM | 22 | Autocorrelation, Joint Average, Cluster Prominence, Cluster Shade, Cluster Tendency, Contrast, Correlation, Difference Average, Difference Entropy, Difference Variance, Joint Energy, Joint Entropy, Informational Measure of Correlation 1, Informational Measure of Correlation 2, Inverse Difference Moment, Inverse Difference Moment Normalized, Inverse Difference, Inverse Difference Normalized, Inverse Variance, Maximum Probability, Sum Entropy, Sum of Squares |
|  | GLRLM | 16 | Short Run Emphasis, Long Run Emphasis, Gray Level Non-Uniformity, Gray Level Non-Uniformity Normalized, Run Length Non-Uniformity, Run Length Non-Uniformity Normalized, Run Percentage, Gray Level Variance, Run Variance, Run Entropy, Low Gray Level Run Emphasis, High Gray Level Run Emphasis, Short Run Low Gray Level Emphasis, Short Run High Gray Level Emphasis, Long Run Low Gray Level Emphasis, Long Run High Gray Level Emphasis |
|  | GLSZM | 16 | Small Area Emphasis, Large Area Emphasis, Gray Level Non-Uniformity, Gray Level Non-Uniformity Normalized, Size-Zone Non-Uniformity, Size-Zone Non-Uniformity Normalized, Zone Percentage, Gray Level Variance, Zone Variance, Zone Entropy, Low Gray Level Zone Emphasis, High Gray Level Zone Emphasis, Small Area Low Gray Level Emphasis, Small Area High Gray Level Emphasis, Large Area Low Gray Level Emphasis, Large Area High Gray Level Emphasis |
|  | GLDM | 14 | Small Dependence Emphasis, Large Dependence Emphasis, Gray Level Non-Uniformity, Dependence Non-Uniformity, Dependence Non-Uniformity Normalized, Gray Level Variance, Dependence Variance, Dependence Entropy, Low Gray Level Emphasis, High Gray Level Emphasis, Small Dependence Low Gray Level Emphasis, Small Dependence High Gray Level Emphasis, Large Dependence Low Gray Level Emphasis, Large Dependence High Gray Level Emphasis |
|  | NGTDM | 5 | Coarseness, Contrast, Busyness, Complexity, Strength |
| Filters | Wavelet | 91*8 | First order features + Texture features |

Note: NF, Number of features; GLCM, gray-level cooccurrence matrix, GLRLM, gray-level run-length matrix, GLSZM, gray-level size zone matrix, NGTDM, neighborhood gray-tone difference matrix; GLDM, gray-level dependence matrix.

Table S2. The selected combination of radiomics features derived from hippocampal ALFF, DC and ReHo.

|  | Feature | Weight coefficients |
| --- | --- | --- |
| ALFF |  |  |
| 1 | wavelet-HHH_glszm_GrayLevelNonUniformity | -0.0303 |
| 2 | wavelet-HHL_glcm_Idmn | -0.0222 |
| 3 | wavelet-HHL_glcm_Idn | -0.0125 |
| 4 | wavelet-HLH_gldm_SmallDependenceLowGrayLevelEmphasis | -0.0125 |
| 5 | wavelet-HLL_firstorder_Skewness | -0.0398 |
| 6 | wavelet-LLL_glcm_Idn | -0.0064 |
| 7 | wavelet-LLL_ngtdm_Coarseness | -0.0392 |
| DC |  |  |
| 1 | wavelet-HHL_firstorder_Median | -0.0132 |
| 2 | wavelet-HHL_glszm_LargeAreaHighGrayLevelEmphasis | -0.0370 |
| 3 | wavelet-HLH_firstorder_Skewness | 0.0138 |
| 4 | wavelet-HLH_gldm_DependenceEntropy | -0.0341 |
| 5 | wavelet-HLH_glszm_LargeAreaHighGrayLevelEmphasis | -0.0141 |
| 6 | wavelet-HLH_glszm_SmallAreaLowGrayLevelEmphasis | 0.0444 |
| 7 | wavelet-HLH_ngtdm_Contrast | -0.0178 |
| 8 | wavelet-HLL_glcm_ClusterShade | 0.0068 |
| 9 | wavelet-LHH_firstorder_Kurtosis | 0.0247 |
| 10 | wavelet-LHH_glcm_Imc1 | 0.0151 |
| 11 | wavelet-LHH_glszm_SmallAreaLowGrayLevelEmphasis | -0.0461 |
| 12 | wavelet-LHL_firstorder_TotalEnergy | -0.0410 |
| ReHo |  |  |
| 1 | wavelet-LLL_firstorder_Minimum | 0.0268 |
| 2 | wavelet-HLH_firstorder_TotalEnergy | 0.0814 |
| 3 | wavelet-HLH_glcm_Idn | 0.0127 |
| 4 | wavelet-HLH_ngtdm_Coarseness | -0.0109 |
| 5 | wavelet-HLL_glszm_LargeAreaHighGrayLevelEmphasis | 0.0496 |
| 6 | wavelet-HLL_glszm_SmallAreaLowGrayLevelEmphasis | 0.0104 |
| 7 | wavelet-LHH_firstorder_Median | 0.0073 |
| 8 | wavelet-LLH_ngtdm_Coarseness | 0.0096 |
| 9 | wavelet-LLH_ngtdm_Strength | 0.0040 |
| 10 | wavelet-LLL_firstorder_10Percentile | 0.0760 |
| 11 | wavelet-LLL_firstorder_Kurtosis | -0.0338 |
| 12 | wavelet-LLL_firstorder_Minimum | 0.0261 |
| 13 | wavelet-LLL_glszm_SmallAreaLowGrayLevelEmphasis | 0.0924 |

Note: glrlm, gray-level run-length matrix, glszm, gray-level size zone matrix, ngtdm, neighborhood gray-tone difference matrix; gldm, gray-level dependence matrix.

Table S3. The selected combination of radiomics features derived from hippocampal ALFF, DC

and VMHC .

|  | Feature | Weight coefficients |
| --- | --- | --- |
| ALFF |  |  |
| 1 | wavelet-HHH_glszm_GrayLevelNonUniformity | -0.0323 |
| 2 | wavelet-HHL_glcm_Idmn | -0.0058 |
| 3 | wavelet-HHL_glcm_Idn | -0.0002 |
| 4 | wavelet-HLH_gldm_SmallDependenceLowGrayLevelEmphasis | -0.0656 |
| 5 | wavelet-HLL_firstorder_Skewness | -0.0656 |
| DC |  |  |
| 1 | wavelet-HHL_glszm_LargeAreaHighGrayLevelEmphasis | -0.0211 |
| 2 | wavelet-HHL_ngtdm_Coarseness | 0.0076 |
| 3 | wavelet-HLH_gldm_DependenceEntropy | -0.0565 |
| 4 | wavelet-HLH_glszm_SmallAreaLowGrayLevelEmphasis | 0.0137 |
| 5 | wavelet-HLH_ngtdm_Contrast | -0.0294 |
| 6 | wavelet-LHH_firstorder_Kurtosis | 0.0358 |
| 7 | wavelet-LHH_glcm_Imc1 | 0.0221 |
| 8 | wavelet-LHH_glszm_SizeZoneNonUniformityNormalized | 0.0008 |
| 9 | wavelet-LHH_glszm_SmallAreaLowGrayLevelEmphasis | 0.0008 |
| 10 | wavelet-LHL_firstorder_Skewness | -0.0177 |
| 11 | wavelet-LHL_firstorder_TotalEnergy | -0.0433 |
| VMHC |  |  |
| 1 | original_glszm_LargeAreaHighGrayLevelEmphasis | 0.0223 |
| 2 | wavelet-HHH_gldm_GrayLevelVariance | -0.0655 |
| 3 | wavelet-HHH_glszm_SizeZoneNonUniformity | 0.0092 |
| 4 | wavelet-HLH_glcm_ClusterShade | 0.0092 |
| 5 | wavelet-HLH_gldm_SmallDependenceLowGrayLevelEmphasis | 0.0122 |
| 6 | wavelet-HLH_glszm_SizeZoneNonUniformityNormalized | 0.0584 |
| 7 | wavelet-HLL_glcm_Correlation | -0.0960 |
| 8 | wavelet-HLL_glcm_Idn | -0.0030 |
| 9 | wavelet-LHH_gldm_DependenceEntropy | 0.0597 |
| 10 | wavelet-LHH_glszm_SmallAreaLowGrayLevelEmphasis | -0.0333 |
| 11 | wavelet-LHL_glszm_Gray Level NonUniformity | 0.0168 |
| 12 | wavelet-LLH_gldm_SmallDependenceLowGrayLevelEmphasis | -0.0030 |
| 13 | wavelet-LLH_ngtdm_Strength | 0.0168 |
| 14 | wavelet-LLL_firstorder_TotalEnergy | 0.0137 |
| 15 | wavelet-LLL_glcm_Correlation | -0.0198 |
| 16 | wavelet-LLL_ngtdm_Busyness | 0.0045 |

Note: glrlm, gray-level run-length matrix, glszm, gray-level size zone matrix, ngtdm, neighborhood gray-tone difference matrix; gldm, gray-level dependence matrix.

Table S4. The selected combination of radiomics features derived from hippocampal ALFF, ReHo and VMHC.

|  | Feature | Weight coefficients |
| --- | --- | --- |
| ALFF |  |  |
| 1 | wavelet-HHH_glszm_GrayLevelNonUniformity | -0.0252 |
| 2 | wavelet-HHL_glcm_Idmn | -0.0097 |
| 3 | wavelet-HHL_glcm_Idn | -0.0434 |
| 4 | wavelet-HHL_glszm_LargeAreaHighGrayLevelEmphasis | -0.0061 |
| 5 | wavelet-HLH_gldm_SmallDependenceLowGrayLevelEmphasis | -0.0899 |
| 6 | wavelet-HLH_ngtdm_Coarseness | -0.0123 |
| 7 | wavelet-HLL_firstorder_Skewness | -0.0192 |
| ReHo |  |  |
| 1 | wavelet-HHH_glszm_SmallAreaEmphasis | 0.0118 |
| 2 | wavelet-HHL_glszm_GrayLevelNonUniformity | 0.0043 |
| 3 | wavelet-HHL_glszm_LargeAreaHighGrayLevelEmphasis | -0.0056 |
| 4 | wavelet-HLH_firstorder_Skewness | 0.0045 |
| 5 | wavelet-HLH_firstorder_TotalEnergy | 0.0495 |
| 6 | wavelet-HLL_glszm_LargeAreaHighGrayLevelEmphasis | 0.0368 |
| 7 | wavelet-LHH_ngtdm_Contrast | -0.0099 |
| 8 | wavelet-LLH_glszm_Gray Level NonUniformity | -0.0395 |
| 9 | wavelet-LLL_firstorder_10Percentile | 0.0763 |
| 10 | wavelet-LLL_firstorder_Kurtosis | -0.0311 |
| 11 | wavelet-LLL_firstorder_Minimum | 0.0005 |
| 12 | wavelet-LLL_glszm_SmallAreaLowGrayLevelEmphasis | 0.0901 |
| VMHC |  |  |
| 1 | wavelet-HHH_gldm_GrayLevelVariance | -0.0401 |
| 2 | wavelet-HHH_glrlm_ShortRunHighGrayLevelEmphasis | -0.0347 |
| 3 | wavelet-HHH_glszm_SizeZoneNonUniformityNormalized | 0.0203 |
| 4 | wavelet-HHL_firstorder_Kurtosis | -0.0061 |
| 5 | wavelet-HLH_glcm_ClusterShade | -0.0419 |
| 6 | wavelet-HLH_glszm_SizeZoneNonUniformityNormalized | 0.0571 |
| 7 | wavelet-HLL_firstorder_Kurtosis | -0.0054 |
| 8 | wavelet-HLL_glcm_Correlation | -0.0780 |
| 9 | wavelet-LHH_gldm_DependenceEntropy | 0.0178 |
| 10 | wavelet-LHH_glszm_SmallAreaLowGrayLevelEmphasis | -0.0037 |
| 11 | wavelet-LHL_firstorder_10Percentile | -0.0010 |
| 12 | wavelet-LHL_firstorder_TotalEnergy | -0.0122 |
| 13 | wavelet-LHL_glszm_Gray Level NonUniformity | 0.0503 |
| 14 | wavelet-LLH_gldm_SmallDependenceLowGrayLevelEmphasis | -0.0299 |
| 15 | wavelet-LLH_ngtdm_Strength | 0.0015 |
| 16 | wavelet-LLL_firstorder_TotalEnergy | 0.0038 |

Note: glrlm, gray-level run-length matrix, glszm, gray-level size zone matrix, ngtdm, neighborhood gray-tone difference matrix; gldm, gray-level dependence matrix.

Table S5. The selected combination of radiomics features derived from hippocampal DC, ReHo and VMHC.

|  | Feature | Weight coefficients |
| --- | --- | --- |
| DC |  |  |
| 1 | wavelet-HHL_glszm_LargeAreaHighGrayLevelEmphasis | -0.0118 |
| 2 | wavelet-HHL_ngtdm_Coarseness | 0.0002 |
| 3 | wavelet-HLH_firstorder_Skewness | 0.0100 |
| 4 | wavelet-HLH_gldm_DependenceEntropy | -0.0477 |
| 5 | wavelet-HLH_glszm_SmallAreaLowGrayLevelEmphasis | 0.0147 |
| 6 | wavelet-LHH_firstorder_Kurtosis | 0.0395 |
| 7 | wavelet-LHH_glcm_Imc1 | 0.0031 |
| 8 | wavelet-LHH_glszm_SmallAreaLowGrayLevelEmphasis | -0.0620 |
| 9 | wavelet-LHL_firstorder_Skewness | -0.0047 |
| 10 | wavelet-LHL_firstorder_TotalEnergy | -0.0813 |
| ReHo |  |  |
| 1 | wavelet-HLH_firstorder_TotalEnergy | 0.0764 |
| 2 | wavelet-HLL_glszm_LargeAreaHighGrayLevelEmphasis | 0.0440 |
| 3 | wavelet-LHH_ngtdm_Contrast | -0.0121 |
| 4 | wavelet-LLH_glszm_Gray Level NonUniformity | -0.0103 |
| 5 | wavelet-LLL_firstorder_10Percentile | 0.0526 |
| 6 | wavelet-LLL_firstorder_Minimum | 0.0309 |
| 7 | wavelet-LLL_glszm_SmallAreaLowGrayLevelEmphasis | 0.1027 |
| VMHC |  |  |
| 1 | wavelet-HHH_gldm_GrayLevelVariance | -0.0562 |
| 2 | wavelet-HHH_glrlm_ShortRunHighGrayLevelEmphasis | -0.0041 |
| 3 | wavelet-HHL_glszm_SmallAreaEmphasis | -0.0102 |
| 4 | wavelet-HLH_glcm_ClusterShade | -0.0274 |
| 5 | wavelet-HLH_glszm_SizeZoneNonUniformityNormalized | 0.0274 |
| 6 | wavelet-HLL_firstorder_Kurtosis | -0.0012 |
| 7 | wavelet-HLL_glcm_Correlation | -0.0740 |
| 8 | wavelet-LHH_gldm_DependenceEntropy | 0.0402 |
| 9 | wavelet-LHH_glszm_SmallAreaLowGrayLevelEmphasis | -0.0274 |
| 10 | wavelet-LHL_firstorder_TotalEnergy | -0.0001 |
| 11 | wavelet-LHL_glszm_Gray Level NonUniformity | 0.0648 |
| 12 | wavelet-LLH_ngtdm_Coarseness | -0.0154 |
| 13 | wavelet-LLL_firstorder_TotalEnergy | 0.0262 |

Note: glrlm, gray-level run-length matrix, glszm, gray-level size zone matrix, ngtdm, neighborhood gray-tone difference matrix; gldm, gray-level dependence matrix..

Table S6. The selected combination of radiomics features derived from hippocampal ALFF and DC.

|  | Feature | Weight coefficients |
| --- | --- | --- |
| ALFF |  |  |
| 1 | wavelet-HHH_glszm_GrayLevelNonUniformity | -0.0555 |
| 2 | wavelet-HHL_glcm_Idmn | -0.0302 |
| 3 | wavelet-HHL_glcm_Idn | 0.0074 |
| 4 | wavelet-HLH_gldm_SmallDependenceLowGrayLevelEmphasis | -0.058 |
| 5 | wavelet-HLL_firstorder_Skewness | -0.0637 |
| 6 | wavelet-HLL_glcm_ClusterShade | -0.0182 |
| 7 | wavelet-LLH_glcm_ClusterShade | 0.0276 |
| 8 | wavelet-LLH_glszm_SmallAreaLowGrayLevelEmphasis | -0.001 |
| 9 | wavelet-LLL_firstorder_Median | 0.0143 |
| 10 | wavelet-LLL_glszm_LowGrayLevelZoneEmphasis | -0.0063 |
| 11 | wavelet-LLL_ngtdm_Coarseness | -0.0199 |
| DC |  |  |
| 1 | wavelet-HHH_gldm_DependenceVariance | 0.0157 |
| 2 | wavelet-HHL_firstorder_Kurtosis | -0.0125 |
| 3 | wavelet-HHL_firstorder_Median | -0.0123 |
| 4 | wavelet-HHL_glszm_LargeAreaHighGrayLevelEmphasis | -0.036 |
| 5 | wavelet-HLH_firstorder_Skewness | 0.0206 |
| 6 | wavelet-HLH_gldm_DependenceEntropy | -0.035 |
| 7 | wavelet-HLH_glszm_SmallAreaLowGrayLevelEmphasis | 0.0404 |
| 8 | wavelet-HLH_ngtdm_Contrast | -0.0573 |
| 9 | wavelet-HLL_glcm_ClusterShade | 0.0624 |
| 10 | wavelet-HLL_glszm_GrayLevelNonUniformity | -0.0039 |
| 11 | wavelet-LHH_firstorder_Kurtosis | -0.0039 |
| 12 | wavelet-LHH_glcm_Imc1 | 0.0462 |
| 13 | wavelet-LHH_glszm_GrayLevelNonUniformity | 0.0383 |
| 14 | wavelet-LHH_glszm_SmallAreaLowGrayLevelEmphasis | -0.0389 |
| 15 | wavelet-LHL_firstorder_Skewness | -0.0253 |
| 16 | wavelet-LHL_firstorder_TotalEnergy | -0.0238 |
| 17 | wavelet-LLL_firstorder_Minimum | 0.034 |
| 18 | wavelet-LLL_glcm_Idn | -0.0192 |

Note: glrlm, gray-level run-length matrix, glszm, gray-level size zone matrix, ngtdm, neighborhood gray-tone difference matrix; gldm, gray-level dependence matrix.

Table S7. The selected combination of radiomics features derived from hippocampal ALFF and ReHo.

|  | Feature | Weight coefficients |
| --- | --- | --- |
| ALFF |  |  |
| 1 | wavelet-HHH_glszm_GrayLevelNonUniformity | -0.0170 |
| 2 | wavelet-HHH_glszm_SmallAreaEmphasis | -0.0267 |
| 3 | wavelet-HHL_glcm_Idmn | -0.0027 |
| 4 | wavelet-HHL_glcm_Idn | -0.0862 |
| 5 | wavelet-HHL_gldm_SmallDependenceLowGrayLevelEmphasis | 0.0114 |
| 6 | wavelet-HHL_glszm_LargeAreaHighGrayLevelEmphasis | -0.0006 |
| 7 | wavelet-HHL_ngtdm_Contrast | 0.0003 |
| 8 | wavelet-HLH_firstorder_Skewness | 0.0050 |
| 9 | wavelet-HLH_glcm_Correlation | -0.0136 |
| 10 | wavelet-HLH_gldm_SmallDependenceLowGrayLevelEmphasis | -0.0700 |
| 11 | wavelet-HLL_firstorder_Skewness | -0.0122 |
| 12 | wavelet-LHL_glrlm_ShortRunLowGrayLevelEmphasis | -0.0214 |
| 13 | wavelet-LLL_ngtdm_Coarseness | -0.0231 |
| ReHo |  |  |
| 1 | wavelet-HHH_gldm_SmallDependenceEmphasis | 0.0179 |
| 2 | wavelet-HHH_gldm_SmallDependenceLowGrayLevelEmphasis | 0.0016 |
| 3 | wavelet-HHL_glszm_LargeAreaHighGrayLevelEmphasis | -0.0149 |
| 4 | wavelet-HLH_firstorder_TotalEnergy | 0.0717 |
| 5 | wavelet-HLH_ngtdm_Coarseness | -0.0188 |
| 6 | wavelet-HLL_glszm_LargeAreaHighGrayLevelEmphasis | 0.0494 |
| 7 | wavelet-HLL_glszm_SmallAreaLowGrayLevelEmphasis | 0.0091 |
| 8 | wavelet-LHH_ngtdm_Contrast | -0.0195 |
| 9 | wavelet-LLH_glszm_Gray Level NonUniformity | -0.0165 |
| 10 | wavelet-LLH_ngtdm_Coarseness | 0.0595 |
| 11 | wavelet-LLL_firstorder_10Percentile | 0.1082 |
| 12 | wavelet-LLL_firstorder_Kurtosis | -0.0616 |
| 13 | wavelet-LLL_firstorder_Minimum | 0.0063 |
| 14 | wavelet-LLL_glszm_SmallAreaLowGrayLevelEmphasis | 0.1048 |

Note: glrlm, gray-level run-length matrix, glszm, gray-level size zone matrix, ngtdm, neighborhood gray-tone difference matrix; gldm, gray-level dependence matrix.

Table S8. The selected combination of radiomics features derived from hippocampal ALFF and VMHC .

|  | Feature | Weight coefficients |
| --- | --- | --- |
| ALFF |  |  |
| 1 | wavelet-HHH_glszm_GrayLevelNonUniformity | -0.0513 |
| 2 | wavelet-HHL_glcm_Idmn | -0.0119 |
| 3 | wavelet-HHL_glcm_Idn | -0.0448 |
| 4 | wavelet-HLH_glcm_Correlation | -0.0040 |
| 5 | wavelet-HLH_gldm_SmallDependenceLowGrayLevelEmphasis | -0.0961 |
| 6 | wavelet-HLL_firstorder_Skewness | -0.0432 |
| 7 | wavelet-LHH_firstorder_Median | 0.0072 |
| 8 | wavelet-LLL_glcm_JointEntropy | -0.0110 |
| VMHC |  |  |
| 1 | original_glszm_LargeAreaHighGrayLevelEmphasis | 0.0246 |
| 2 | wavelet-HHH_gldm_GrayLevelVariance | -0.0525 |
| 3 | wavelet-HHH_glrlm_ShortRunHighGrayLevelEmphasis | -0.0293 |
| 4 | wavelet-HHH_glszm_SizeZoneNonUniformity | 0.0450 |
| 5 | wavelet-HHL_firstorder_Kurtosis | -0.0073 |
| 6 | wavelet-HLH_glcm_ClusterShade | -0.0444 |
| 7 | wavelet-HLH_glszm_LowGrayLevelZoneEmphasis | 0.0037 |
| 8 | wavelet-HLH_glszm_SizeZoneNonUniformityNormalized | 0.0776 |
| 9 | wavelet-HLL_firstorder_Kurtosis | -0.0156 |
| 10 | wavelet-HLL_glcm_Correlation | -0.0156 |
| 11 | wavelet-LHH_gldm_DependenceEntropy | 0.0475 |
| 12 | wavelet-LHH_glszm_SmallAreaLowGrayLevelEmphasis | -0.0105 |
| 13 | wavelet-LHL_glszm_Gray Level NonUniformity | 0.0177 |
| 14 | wavelet-LLH_gldm_SmallDependenceLowGrayLevelEmphasis | -0.0341 |
| 15 | wavelet-LLH_ngtdm_Strength | 0.0278 |
| 16 | wavelet-LLL_firstorder_TotalEnergy | 0.0005 |
| 17 | wavelet-LLL_glcm_Correlation | 0.0005 |
| 18 | wavelet-LLL_glszm_ZoneVariance | 0.0138 |

Note: glrlm, gray-level run-length matrix, glszm, gray-level size zone matrix, ngtdm, neighborhood gray-tone difference matrix; gldm, gray-level dependence matrix.

Table S9. The selected combination of radiomics features derived from hippocampal DC and ReHo.

|  | Feature | Weight coefficients |
| --- | --- | --- |
| DC |  |  |
| 1 | wavelet-HHL_firstorder_Median | -0.0134 |
| 2 | wavelet-HHL_glszm_LargeAreaHighGrayLevelEmphasis | -0.0134 |
| 3 | wavelet-HHL_ngtdm_Coarseness | 0.0055 |
| 4 | wavelet-HLH_firstorder_Skewness | 0.0165 |
| 5 | wavelet-HLH_gldm_DependenceEntropy | -0.0299 |
| 6 | wavelet-HLH_glszm_SmallAreaLowGrayLevelEmphasis | 0.0394 |
| 7 | wavelet-HLH_ngtdm_Contrast | -0.0232 |
| 8 | wavelet-HLL_glszm_GrayLevelNonUniformity | -0.0182 |
| 9 | wavelet-LHH_firstorder_Kurtosis | 0.0292 |
| 10 | wavelet-LHH_glcm_Imc1 | 0.0093 |
| 11 | wavelet-LHH_glszm_SmallAreaLowGrayLevelEmphasis | 0.0576 |
| 12 | wavelet-LHH_ngtdm_Contrast | 0.0080 |
| 13 | wavelet-LHL_firstorder_TotalEnergy | 0.0080 |
| 14 | wavelet-LLL_firstorder_Minimum | 0.0296 |
| ReHo |  |  |
| 1 | wavelet-HHH_firstorder_Kurtosis | 0.0296 |
| 2 | wavelet-HLH_firstorder_Median | 0.0045 |
| 3 | wavelet-HLH_firstorder_TotalEnergy | 0.0923 |
| 4 | wavelet-HLH_ngtdm_Coarseness | -0.0253 |
| 5 | wavelet-HLL_glszm_LargeAreaHighGrayLevelEmphasis | 0.0575 |
| 6 | wavelet-HLL_glszm_SmallAreaLowGrayLevelEmphasis | 0.0124 |
| 7 | wavelet-LHH_firstorder_Median | 0.0067 |
| 8 | wavelet-LHH_ngtdm_Contrast | -0.0319 |
| 9 | wavelet-LLH_ngtdm_Coarseness | 0.0146 |
| 10 | wavelet-LLH_ngtdm_Strength | 0.0146 |
| 11 | wavelet-LLL_firstorder_10Percentile | 0.0926 |
| 12 | wavelet-LLL_firstorder_Kurtosis | -0.0384 |
| 13 | wavelet-LLL_firstorder_Minimum | 0.0141 |
| 14 | wavelet-LLL_glszm_SmallAreaLowGrayLevelEmphasis | 0.1007 |

Note: glrlm, gray-level run-length matrix, glszm, gray-level size zone matrix, ngtdm, neighborhood gray-tone difference matrix; gldm, gray-level dependence matrix.

Table S10. The selected combination of radiomics features derived from hippocampal DC and VMHC.

|  | Feature | Weight coefficients |
| --- | --- | --- |
| DC |  |  |
| 1 | wavelet-HHL_glszm_LargeAreaHighGrayLevelEmphasis | -0.0272 |
| 2 | wavelet-HHL_ngtdm_Coarseness | 0.0319 |
| 3 | wavelet-HLH_gldm_DependenceEntropy | -0.0498 |
| 4 | wavelet-HLH_glszm_SmallAreaLowGrayLevelEmphasis | 0.0000 |
| 5 | wavelet-HLH_ngtdm_Contrast | 0.0000 |
| 6 | wavelet-HLL_glszm_GrayLevelNonUniformity | -0.0159 |
| 7 | wavelet-LHH_firstorder_Kurtosis | 0.0576 |
| 8 | wavelet-LHH_glcm_Imc1 | 0.0297 |
| 9 | wavelet-LHH_glszm_SizeZoneNonUniformityNormalized | 0.0115 |
| 10 | wavelet-LHH_glszm_SmallAreaLowGrayLevelEmphasis | -0.0637 |
| 11 | wavelet-LHL_firstorder_Skewness | -0.0637 |
| 12 | wavelet-LHL_firstorder_TotalEnergy | -0.0717 |
| 13 | wavelet-LHL_glcm_ClusterShade | -0.0014 |
| 14 | wavelet-LLH_firstorder_Median | 0.0142 |
| VMHC |  |  |
| 1 | original_glszm_LargeAreaHighGrayLevelEmphasis | 0.0206 |
| 2 | wavelet-HHH_gldm_GrayLevelVariance | -0.0781 |
| 3 | wavelet-HHH_glszm_SizeZoneNonUniformity | 0.0091 |
| 4 | wavelet-HLH_glcm_ClusterShade | -0.0162 |
| 5 | wavelet-HLH_glszm_LowGrayLevelZoneEmphasis | 0.0255 |
| 6 | wavelet-HLH_glszm_SizeZoneNonUniformityNormalized | 0.0351 |
| 7 | wavelet-HLL_glcm_ClusterShade | 0.0042 |
| 8 | wavelet-HLL_glcm_Correlation | -0.0870 |
| 9 | wavelet-HLL_glcm_Idn | -0.0070 |
| 10 | wavelet-LHH_gldm_DependenceEntropy | 0.0685 |
| 11 | wavelet-LHH_glszm_SmallAreaLowGrayLevelEmphasis | -0.0543 |
| 12 | wavelet-LHL_firstorder_TotalEnergy | -0.0048 |
| 13 | wavelet-LHL_glszm_Gray Level NonUniformity | 0.0095 |
| 14 | wavelet-LLH_gldm_SmallDependenceLowGrayLevelEmphasis | -0.0054 |
| 15 | wavelet-LLL_firstorder_TotalEnergy | 0.0145 |
| 16 | wavelet-LLL_glcm_Correlation | -0.0305 |
| 17 | wavelet-LLL_ngtdm_Busyness | 0.0046 |

Note: glrlm, gray-level run-length matrix, glszm, gray-level size zone matrix, ngtdm, neighborhood gray-tone difference matrix; gldm, gray-level dependence matrix.

Table S11. The selected combination of radiomics features derived from hippocampal ReHo and VMHC.

|  | Feature | Weight coefficients |
| --- | --- | --- |
| ReHo |  |  |
| 1 | wavelet-HHH_gldm_DependenceVariance | 0.0311 |
| 2 | wavelet-HHH_gldm_SmallDependenceLowGrayLevelEmphasis | 0.0018 |
| 3 | wavelet-HHH_glszm_SmallAreaEmphasis | 0.0155 |
| 4 | wavelet-HHL_glszm_GrayLevelNonUniformity | 0.0006 |
| 5 | wavelet-HLH_firstorder_TotalEnergy | 0.0708 |
| 6 | wavelet-HLH_ngtdm_Coarseness | -0.0090 |
| 7 | wavelet-HLL_glszm_LargeAreaHighGrayLevelEmphasis | 0.0331 |
| 8 | wavelet-LHH_ngtdm_Contrast | -0.0141 |
| 9 | wavelet-LLH_glszm_Gray Level NonUniformity | -0.0531 |
| 10 | wavelet-LLL_firstorder_10Percentile | 0.0715 |
| 11 | wavelet-LLL_firstorder_Kurtosis | -0.0178 |
| 12 | wavelet-LLL_firstorder_Minimum | 0.0098 |
| 13 | wavelet-LLL_glszm_SmallAreaLowGrayLevelEmphasis | 0.0998 |
| VMHC |  |  |
| 1 | wavelet-HHH_gldm_GrayLevelVariance | -0.0324 |
| 2 | wavelet-HHH_glrlm_ShortRunHighGrayLevelEmphasis | -0.0456 |
| 3 | wavelet-HHH_glszm_SizeZoneNonUniformityNormalized | 0.0285 |
| 4 | wavelet-HHL_firstorder_Kurtosis | -0.0019 |
| 5 | wavelet-HHL_glszm_SmallAreaEmphasis | -0.0127 |
| 6 | wavelet-HLH_glcm_ClusterShade | -0.0331 |
| 7 | wavelet-HLH_glszm_SizeZoneNonUniformityNormalized | 0.0350 |
| 8 | wavelet-HLL_firstorder_Kurtosis | -0.0252 |
| 9 | wavelet-HLL_glcm_Correlation | -0.0897 |
| 10 | wavelet-LHH_gldm_DependenceEntropy | 0.0073 |
| 11 | wavelet-LHH_glszm_SmallAreaLowGrayLevelEmphasis | -0.0336 |
| 12 | wavelet-LHL_firstorder_TotalEnergy | -0.0349 |
| 13 | wavelet-LHL_glszm_Gray Level NonUniformity | 0.0415 |
| 14 | wavelet-LLH_gldm_SmallDependenceLowGrayLevelEmphasis | -0.0272 |
| 15 | wavelet-LLH_ngtdm_Coarseness | -0.0225 |
| 16 | wavelet-LLH_ngtdm_Contrast | 0.0135 |
| 17 | wavelet-LLL_glszm_LowGrayLevelZoneEmphasis | -0.0208 |

Note: glrlm, gray-level run-length matrix, glszm, gray-level size zone matrix, ngtdm, neighborhood gray-tone difference matrix; gldm, gray-level dependence matrix.

Table S12. The selected radiomics features derived from hippocampal ALFF.

|  | Feature | Weight coefficients |
| --- | --- | --- |
| 1 | wavelet-HHH_glszm_GrayLevelNonUniformity | -0.0506 |
| 2 | wavelet-HHL_glcm_Idmn | -0.0733 |
| 3 | wavelet-HLH_firstorder_Skewness | 0.0041 |
| 4 | wavelet-HLH_glcm_Correlation | -0.0571 |
| 5 | wavelet-HLH_gldm_SmallDependenceLowGrayLevelEmphasis | -0.1127 |
| 6 | wavelet-HLL_firstorder_Skewness | -0.0688 |
| 7 | wavelet-HLL_glszm_SmallAreaLowGrayLevelEmphasis | 0.0161 |
| 8 | wavelet-LHL_firstorder_Kurtosis | -0.0109 |
| 9 | wavelet-LHL_glrlm_ShortRunLowGrayLevelEmphasis | -0.0434 |
| 10 | wavelet-LLH_glcm_ClusterShade | 0.3344 |
| 11 | wavelet-LLH_glszm_SmallAreaLowGrayLevelEmphasis | -0.0170 |
| 12 | wavelet-LLL_firstorder_Median | 0.0241 |
| 13 | wavelet-LLL_glszm_LowGrayLevelZoneEmphasis | -0.0191 |
| 14 | wavelet-LLL_ngtdm_Coarseness | -0.0220 |

Note: glrlm, gray-level run-length matrix, glszm, gray-level size zone matrix, ngtdm, neighborhood gray-tone difference matrix; gldm, gray-level dependence matrix.

Table S13. The selected radiomics features derived from hippocampal DC.

|  | Feature | Weight coefficients |
| --- | --- | --- |
| 1 | wavelet-HHH_gldm_DependenceVariance | 0.0245 |
| 2 | wavelet-HHL_firstorder_Kurtosis | -0.0259 |
| 3 | wavelet-HHL_glszm_LargeAreaHighGrayLevelEmphasis | -0.0424 |
| 4 | wavelet-HHL_ngtdm_Coarseness | 0.0146 |
| 5 | wavelet-HLH_firstorder_Skewness | 0.0051 |
| 6 | wavelet-HLH_gldm_DependenceEntropy | -0.0534 |
| 7 | wavelet-HLH_glszm_SmallAreaLowGrayLevelEmphasis | 0.0286 |
| 8 | wavelet-HLH_ngtdm_Contrast | -0.0728 |
| 9 | wavelet-HLL_glcm_ClusterShade | 0.0290 |
| 10 | wavelet-HLL_glszm_GrayLevelNonUniformity | -0.0261 |
| 11 | wavelet-LHH_firstorder_Kurtosis | 0.0821 |
| 12 | wavelet-LHH_glcm_Imc1 | 0.0355 |
| 13 | wavelet-LHH_glszm_GrayLevelNonUniformity | 0.0450 |
| 14 | wavelet-LHH_glszm_SmallAreaLowGrayLevelEmphasis | -0.0360 |
| 15 | wavelet-LHH_ngtdm_Contrast | 0.0282 |
| 16 | wavelet-LHL_firstorder_Skewness | -0.0305 |
| 17 | wavelet-LHL_firstorder_TotalEnergy | -0.0669 |
| 18 | wavelet-LLH_firstorder_TotalEnergy | 0.0071 |
| 19 | wavelet-LLL_firstorder_Minimum | 0.0561 |
| 20 | wavelet-LLL_glcm_Idn | -0.0308 |

Note: glrlm, gray-level run-length matrix, glszm, gray-level size zone matrix, ngtdm, neighborhood gray-tone difference matrix; gldm, gray-level dependence matrix.

Table S14. The selected radiomics features derived from hippocampal ReHo.

|  | Feature | Weight coefficients |
| --- | --- | --- |
| 1 | wavelet-HHH_firstorder_Kurtosis | 0.0054 |
| 2 | wavelet-HHH_gldm_SmallDependenceEmphasis | 0.0203 |
| 3 | wavelet-HHH_gldm_SmallDependenceLowGrayLevelEmphasis | 0.0137 |
| 4 | wavelet-HHL_glszm_LargeAreaHighGrayLevelEmphasis | -0.0136 |
| 5 | wavelet-HLH_firstorder_TotalEnergy | 0.8667 |
| 6 | wavelet-HLH_ngtdm_Coarseness | -0.0284 |
| 7 | wavelet-HLH_ngtdm_Contrast | -0.0091 |
| 8 | wavelet-HLL_glszm_LargeAreaHighGrayLevelEmphasis | 0.0544 |
| 9 | wavelet-HLL_glszm_SmallAreaLowGrayLevelEmphasis | 0.0381 |
| 10 | wavelet-LHH_firstorder_Median | 0.0051 |
| 11 | wavelet-LHH_glszm_SizeZoneNonUniformityNormalized | -0.0029 |
| 12 | wavelet-LHH_ngtdm_Contrast | 0.0665 |
| 13 | wavelet-LLH_glszm_Gray Level NonUniformity | -0.0293 |
| 14 | wavelet-LLH_ngtdm_Coarseness | 0.0283 |
| 15 | wavelet-LLH_ngtdm_Strength | 0.0069 |
| 16 | wavelet-LLL_firstorder_10Percentile | 0.1165 |
| 17 | wavelet-LLL_firstorder_Kurtosis | -0.0670 |
| 18 | wavelet-LLL_firstorder_Minimum | 0.0133 |
| 19 | wavelet-LLL_glszm_SmallAreaLowGrayLevelEmphasis | 0.1166 |

Note: glrlm, gray-level run-length matrix, glszm, gray-level size zone matrix, ngtdm, neighborhood gray-tone difference matrix; gldm, gray-level dependence matrix.

Table S15. The selected radiomics features derived from hippocampal VMHC.

|  | Feature | Weight coefficients |
| --- | --- | --- |
| 1 | wavelet-HHH_gldm_GrayLevelVariance | -0.0572 |
| 2 | wavelet-HHH_glrlm_ShortRunHighGrayLevelEmphasis | -0.0489 |
| 3 | wavelet-HHH_glszm_SizeZoneNonUniformity | 0.0320 |
| 4 | wavelet-HHH_glszm_SizeZoneNonUniformityNormalized | 0.0035 |
| 5 | wavelet-HLH_firstorder_Skewness | -0.0061 |
| 6 | wavelet-HLH_glcm_ClusterShade | -0.0480 |
| 7 | wavelet-HLH_glszm_LowGrayLevelZoneEmphasis | 0.0374 |
| 8 | wavelet-HLH_glszm_SizeZoneNonUniformityNormalized | 0.0453 |
| 9 | wavelet-HLL_firstorder_Kurtosis | -0.0320 |
| 10 | wavelet-HLL_glcm_Correlation | -0.0958 |
| 11 | wavelet-LHH_gldm_DependenceEntropy | 0.0350 |
| 12 | wavelet-LHH_glszm_SmallAreaLowGrayLevelEmphasis | -0.4139 |
| 13 | wavelet-LHH_glszm_ZoneVariance | 0.0209 |
| 14 | wavelet-LHL_firstorder_TotalEnergy | -0.0079 |
| 15 | wavelet-LHL_glszm_Gray Level NonUniformity | 0.0207 |
| 16 | wavelet-LLH_gldm_LargeDependenceHighGrayLevelEmphasis | 0.0251 |
| 17 | wavelet-LLH_gldm_SmallDependenceLowGrayLevelEmphasis | -0.0630 |
| 18 | wavelet-LLH_ngtdm_Coarseness | -0.0141 |
| 19 | wavelet-LLH_ngtdm_Contrast | 0.0682 |
| 20 | wavelet-LLL_glcm_Correlation | -0.0232 |
| 21 | wavelet-LLL_glszm_LowGrayLevelZoneEmphasis | -0.0045 |
| 22 | wavelet-LLL_glszm_ZoneVariance | 0.0105 |

Note: glrlm, gray-level run-length matrix, glszm, gray-level size zone matrix, ngtdm, neighborhood gray-tone difference matrix; gldm, gray-level dependence matrix.

Table S16. The selected radiomics features derived from hippocampal gray matter volume (GMV).

|  | Feature | Weight coefficients |
| --- | --- | --- |
| 1 | wavelet-HHH_glcm_Idn | 0.0192 |
| 2 | wavelet-HHH_glcm_InverseVariance | -0.0489 |
| 3 | wavelet-HHH_gldm_SmallDependenceLowGrayLevelEmphasis | -0.0186 |
| 4 | wavelet-HHH_ngtdm_Busyness | -0.0991 |
| 5 | wavelet-HHL_firstorder_Median | 0.0033 |
| 6 | wavelet-HHL_gldm_SmallDependenceLowGrayLevelEmphasis | -0.0506 |
| 7 | wavelet-HLH_firstorder_Mean | -0.0228 |
| 8 | wavelet-HLH_firstorder_Median | -0.0402 |
| 9 | wavelet-HLL_glszm_ZoneVariance | -0.0822 |
| 10 | wavelet-LHH_firstorder_Skewness | 0.1038 |
| 11 | wavelet-LHH_glszm_ZoneEntropy | 0.0258 |
| 12 | wavelet-LHL_firstorder_Mean | 0.0121 |
| 13 | wavelet-LHL_gldm_SmallDependenceLowGrayLevelEmphasis | 0.0244 |
| 14 | wavelet-LLH_firstorder_Maximum | 0.0041 |
| 15 | wavelet-LLH_glcm_Correlation | -0.0965 |
| 16 | wavelet-LLH_glszm_SmallAreaLowGrayLevelEmphasis | -0.0320 |
| 17 | wavelet-LLH_glszm_ZoneVariance | -0.0018 |
| 18 | wavelet-LLL_firstorder_Minimum | -0.0173 |

Note: glrlm, gray-level run-length matrix, glszm, gray-level size zone matrix, ngtdm, neighborhood gray-tone difference matrix; gldm, gray-level dependence matrix.

Table S17: Prediction performance of the cut-off value from different hippocampal rad-score model for discriminating between CI-PD patients and CP-PD patients.

|  | Training cohort | | | | |  | Validation cohort | | |
| --- | --- | --- | --- | --- | --- | --- | --- | --- | --- |
|  | AUC (95 %CI) | ACC | SPE | SEN | Cut-off |  | ACC | SPE | SEN |
| Modeling of hippocampal radiomics features derived from single imaging metrics | | | | | | | | | |
| ALFF | 0.822(0.712-0.931) | 0.774 | 0.800 | 0.757 | 0.410 |  | 0.704 | 0.556 | 0.778 |
| DC | 0.966(0.924-1.000) | 0.952 | 0.920 | 0.973 | 0.421 |  | 0.703 | 0.778 | 0.667 |
| ReHo | 0.912(0.827-1.000) | 0.887 | 0.920 | 0.865 | 0.440 |  | 0.815 | 0.778 | 0.833 |
| VMHC | 0.903(0.830-0.975) | 0.817 | 0.920 | 0.703 | 0.368 |  | 0.815 | 0.800 | 0.824 |
| Modeling of hippocampal radiomics features derived from two imaging metrics | | | | | | | | | |
| ALFF+DC | 0.869(0.761-0.978) | 0.850 | 0.880 | 0.838 | 0.415 |  | 0.815 | 0.889 | 0.778 |
| ALFF+ReHo | 0.922(0.833-1.000) | 0.919 | 0.880 | 0.946 | 0.474 |  | 0.704 | 0.778 | 1.000 |
| ALFF+VMHC | 0.861(0.764-0.958) | 0.855 | 0.792 | 0.895 | 0.461 |  | 0.852 | 0.800 | 1.000 |
| DC+ReHo | 0.945(0.866-1.000) | 0.919 | 0.96 | 0.892 | 0.432 |  | 0.704 | 1.000 | 0.889 |
| DC+VMHC | 0.831(0.705-0.956) | 0.839 | 0.864 | 0.800 | 0.389 |  | 0.889 | 0.867 | 0.917 |
| ReHo+VMHC* | 0.941(0.886-0.996) | 0.887 | 0.917 | 0.868 | 0.429 |  | 0.889 | 0.900 | 0.882 |
| Modeling of hippocampal radiomics features derived from three imaging metrics | | | | | | | | | |
| ALFF+DC+ReHo | 0.915(0.821-1.000) | 0.887 | 0.909 | 0.875 | 0.422 |  | 0.963 | 1.000 | 0.933 |
| ALFF+DC+VMHC | 0.893(0.807-0.978) | 0.855 | 0.958 | 0.789 | 0.376 |  | 0.852 | 0.800 | 0.882 |
| ALFF+ReHO+VMHC | 0.948(0.893-1.000) | 0.871 | 0.816 | 0.958 | 0.384 |  | 0.889 | 0.800 | 0.941 |
| DC+ReHo+VMHC | 0.889(0.791-0.987) | 0.871 | 0.76 | 0.946 | 0.529 |  | 0.963 | 0.889 | 1.000 |
| Modeling of hippocampal radiomics features derived from four imaging metrics | | | | | | | | | |
| ALFF+DC+ReHo+VMHC | 0.903(0.792-1.00) | 0.919 | 0.913 | 0.923 | 0.441 |  | 0.925 | 0.909 | 0.937 |

Note: AUC, Area Under Curve; ACC, Accuracy; SEN, Sensitivity; SPE, specificity; LR, Logistic Regression; ALFF, Amplitude of Low Frequency Fluctuations; ReHo, Regional Homogeneity; DC, Degree centrality; VMHC, Voxel-Mirrored Homotopic Connectivity. * the optimal combination.


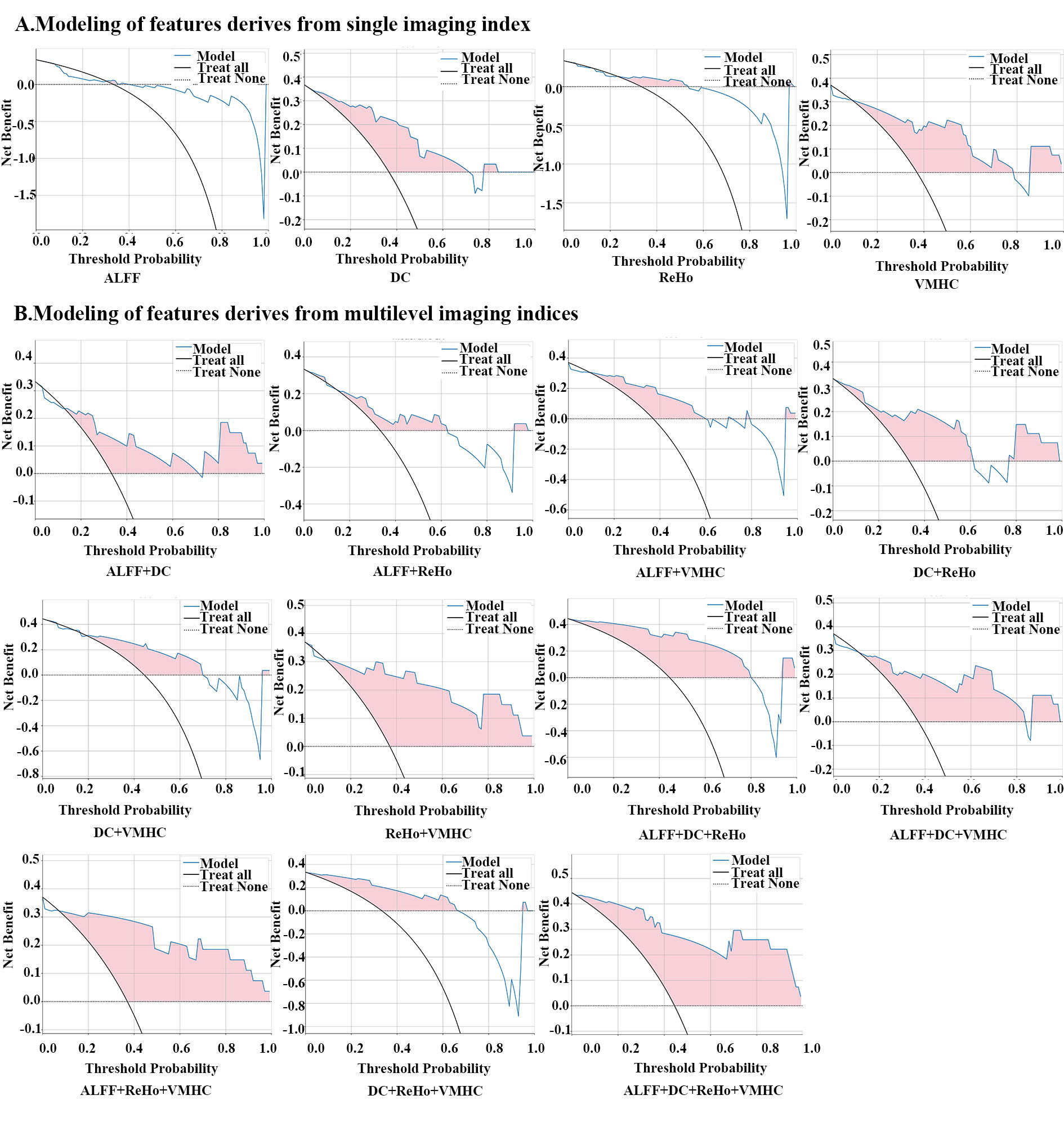


Figure S1. The decision curve analysis (DCA) of the Logistic Regression (LR) models.

Note: ALFF, Amplitude of Low Frequency Fluctuations; ReHo, Regional Homogeneity; DC, Degree centrality; VMHC, Voxel-Mirrored Homotopic Connectivity.

Table S18. Diagnostic performance of radiomics model constructed by the features selected from gray matter volume (GMV) of hippocampus

| GMV | Train | | | | |  | Test | | | |
| --- | --- | --- | --- | --- | --- | --- | --- | --- | --- | --- |
|  | AUC(95 %CI) | ACC | SPE | SEN | Cut-off |  | AUC(95 %CI) | ACC | SPE | SEN |
| Radscore | 0.836(0.731-0.941) | 0.758 | 0.875 | 0.684 | 0.559 |  | - | 0.741 | 0.800 | 0.706 |
| LR model | 0.870(0.776-0.963) | 0.839 | 0.833 | 0.842 | - |  | 0.882(0.740-1.000) | 0.889 | 0.800 | 0.941 |

Note: AUC, Area Under Curve; ACC, Accuracy; SEN, Sensitivity; SPE, specificity.

Table S19 The results of Delong test in different Rad-score models.

| Radscore Models | ALFF | DC | ReHo | VMHC | ALFF  +DC | ALFF  +ReHo | ALFF+  VMHC | DC+  ReHo | DC+  VMHC | ReHo+  VMHC | ALFF  +DC+  ReHo | ALFF  +DC+  VMHC | ALFF+  ReHo+  VMHC | DC+  ReHo+  VMHC | ALFF  +DC+  ReHo+VMHC |
| --- | --- | --- | --- | --- | --- | --- | --- | --- | --- | --- | --- | --- | --- | --- | --- |
| ALFF | - | **0.013^*^** | **0.001^*^** | 0.224 | 0.550 | 0.144 | 0.600 | **0.048^*^** | 0.915 | 0.056 | 0.187 | 0.314 | 0.041 | 0.370 | 0.277 |
| DC | - | - | **0.046^*^** | 0.121 | 0.096 | 0.311 | **0.045^*^** | 0.524 | **0.041^*^** | 0.449 | 0.275 | 0.118 | 0.570 | 0.146 | 0.230 |
| ReHo | - | - | - | 0.877 | 0.547 | 0.868 | 0.445 | 0.533 | 0.299 | 0.584 | 0.962 | 0.761 | 0.490 | 0.732 | 0.893 |
| VMHC | - | - | - | - | 0.610 | 0.726 | 0.496 | 0.364 | 0.328 | 0.411 | 0.833 | 0.860 | 0.321 | 0.821 | 1.000 |
| ALFF+DC | - | - | - | - | - | 0.438 | 0.914 | 0.222 | 0.653 | 0.248 | 0.514 | 0.734 | 0.200 | 0.789 | 0.648 |
| ALFF+ReHo | - | - | - | - | - | - | 0.337 | 0.637 | 0.226 | 0.696 | 0.905 | 0.622 | 0.587 | 0.606 | 0.765 |
| ALFF+VMHC | - | - | - | - | - | - | - | 0.140 | 0.710 | 0.160 | 0.412 | 0.627 | 0.121 | 0.691 | 0.548 |
| DC+ReHo | - | - | - | - | - | - | - | - | 0.102 | 0.920 | 0.561 | 0.314 | 0.938 | 0.329 | 0.460 |
| DC+VMHC | - | - | - | - | - | - | - | - | - | 0.114 | 0.276 | 0.421 | 0.090 | 0.474 | 0.372 |
| ReHo+VMHC | - | - | - | - | - | - | - | - | - | - | 0.615 | 0.353 | 0.856 | 0.364 | 0.504 |
| ALFF+DC+  ReHo | - | - | - | - | - | - | - | - | - | - | - | 0.720 | 0.516 | 0.694 | 0.855 |
| ALFF+DC+  VMHC | - | - | - | - | - | - | - | - | - | - | - | - | 0.279 | 0.952 | 0.879 |
| ALFF+  ReHo+  VMHC | - | - | - | - | - | - | - | - | - | - | - | - | - | 0.764 | 0.928 |
| DC+ ReHo+VMHC | - | - | - | - | - | - | - | - | - | - | - | - | - | - | 0.842 |
| ALFF+DC+ ReHo+VMHC | - | - | - | - | - | - | - | - | - | - | - | - | - | - | - |

Note: * , P ＜0.05; **, P＜0.001; ALFF, Amplitude of Low Frequency Fluctuations; ReHo, Regional Homogeneity; DC, Degree centrality; VMHC, Voxel-Mirrored Homotopic Connectivity.

Table S20. The results of Delong test in different LR models in validation cohort.

| LR Models | ALFF | DC | ReHo | VMHC | ALFF  +DC | ALFF  +ReHo | ALFF+VMHC | DC+  ReHo | DC+  VMHC | ReHo+  VMHC | ALFF  +DC+  ReHo | ALFF  +DC+  VMHC | ALFF+ReHo+VMHC | DC+ ReHo+VMHC | ALFF+DC+ ReHo+VMHC |
| --- | --- | --- | --- | --- | --- | --- | --- | --- | --- | --- | --- | --- | --- | --- | --- |
| ALFF | - | 0.519 | 0.327 | 0.067 | 0.092 | 0.037* | 0.144 | 0.025* | 0.113 | 0.007* | 0.009* | 0.046* | 0.013* | 0.030* | 0.002* |
| DC | - | - | 0.769 | 0.277 | 0.335 | 0.355 | 0.444 | 0.149 | 0.394 | 0.065 | 0.074 | 0.217 | 0.095 | 0.166 | 0.027* |
| ReHo | - | - | - | 0.403 | 0.479 | 0.669 | 0.615 | 0.224 | 0.559 | 0.097 | 0.111 | 0.321 | 0.143 | 0.248 | 0.037* |
| VMHC | - | - | - | - | 0.910 | 0.444 | 0.764 | 0.724 | 0.787 | 0.421 | 0.462 | 0.888 | 0.548 | 0.768 | 0.203 |
| ALFF+DC | - | - | - | - | - | 0.537 | 0.852 | 0.646 | 0.882 | 0.372 | 0.408 | 0.801 | 0.484 | 0.687 | 0.184 |
| ALFF+ReHo | - | - | - | - | - | - | 0.812 | 0.338 | 0.759 | 0.153 | 0.175 | 0.467 | 0.223 | 0.370 | 0.059 |
| ALFF+VMHC | - | - | - | - | - | - | - | 0.522 | 0.961 | 0.293 | 0.322 | 0.662 | 0.384 | 0.558 | 0.146 |
| DC+ReHo | - | - | - | - | - | - | - | - | 0.519 | 0.619 | 0.676 | 0.829 | 0.789 | 0.952 | 0.286 |
| DC+VMHC | - | - | - | - | - | - | - | - | - | 0.265 | 0.297 | 0.674 | 0.366 | 0.559 | 0.112 |
| ReHo+VMHC | - | - | - | - | - | - | - | - | - | - | 0.932 | 0.490 | 0.809 | 0.581 | 0.496 |
| ALFF+DC+ ReHo | - | - | - | - | - | - | - | - | - | - | - | 0.538 | 0.876 | 0.636 | 0.452 |
| ALFF+DC+VMHC | - | - | - | - | - | - | - | - | - | - | - | - | 0.636 | 0.876 | 0.2301 |
| ALFF+ReHo+VMHC | - | - | - | - | - | - | - | - | - | - | - | - | - | 0.745 | 0.381 |
| DC+ ReHo+VMHC | - | - | - | - | - | - | - | - | - | - | - | - | - | - | 0.269 |
| ALFF+DC+ReHo+VMHC | - | - | - | - | - | - | - | - | - | - | - | - | - | - | - |

Note: * , P ＜0.05; **, P＜0.001; ALFF, Amplitude of Low Frequency Fluctuations; ReHo, Regional Homogeneity; DC, Degree centrality; VMHC, Voxel-Mirrored Homotopic Connectivity.

Table S21. The Delong test between Rad-score and LR models.

| Radscore models | LR models | | | | | | | | | | | | | | |
| --- | --- | --- | --- | --- | --- | --- | --- | --- | --- | --- | --- | --- | --- | --- | --- |
|  | ALFF | DC | ReHo | VMHC | ALFF  +DC | ALFF  +ReHo | ALFF+  VMHC | DC+  ReHo | DC+  VMHC | ReHo+  VMHC | ALFF  +DC+  ReHo | ALFF  +DC+  VMHC | ALFF+  ReHo+  VMHC | DC+  ReHo+  VMHC | ALFF  +DC+  ReHo+VMHC |
| ALFF | **0.042 ^*^** | 0.229 | 0.344 | 0.985 | 0.896 | 0.514 | 0.741 | 0.637 | 0.761 | 0.253 | 0.521 | 0.847 | 0.408 | 0.695 | 0.051 |
| DC | **0.001^*^** | **0.016** | **0.020** | 0.121 | 0.920 | **0.032^*^** | 0.092 | 0.154 | 0.062 | 0.239 | 0.170 | 0.132 | 0.192 | 0.150 | 0.510 |
| ReHo | **0.006^*^** | 0.057 | 0.084 | 0.381 | 0.907 | 0.134 | 0.265 | 0.561 | 0.235 | 0.927 | 0.643 | 0.444 | 0.741 | 0.529 | 0.548 |
| VMHC | **0.006^*^** | 0.062 | 0.091 | 0.415 | 0.909 | 0.145 | 0.287 | 0.619 | 0.256 | 0.960 | 0.712 | 0.486 | 0.825 | 0.582 | 0.378 |
| ALFF+DC | **0.018^*^** | 0.124 | 0.186 | 0.670 | 0.891 | 0.288 | 0.473 | 0.955 | 0.462 | 0.614 | 0.934 | 0.776 | 0.812 | 0.905 | 0.230 |
| ALFF+ReHo | **0.004^*^** | **0.046^*^** | 0.066 | 0.318 | 0.910 | 0.105 | 0.223 | 0.468 | 0.190 | 0.797 | 0.538 | 0.370 | 0.625 | 0.441 | 0.653 |
| ALFF+VMHC | **0.019^*^** | 0.132 | 0.199 | 0.718 | 0.889 | 0.309 | 0.505 | 0.972 | 0.496 | 0.514 | 0.852 | 0.833 | 0.721 | 0.973 | 0.146 |
| DC+ReHo | **0.002^*^** | **0.026^*^** | **0.036^*^** | 0.198 | 0.916 | 0.058 | 0.143 | 0.279 | 0.110 | 0.483 | 0.319 | 0.226 | 0.371 | 0.266 | 0.980 |
| DC+VMHC | **0.041^*^** | 0.216 | 0.324 | 0.949 | 0.850 | 0.480 | 0.695 | 0.726 | 0.710 | 0.347 | 0.616 | 0.923 | 0.506 | 0.781 | 0.106 |
| ReHo+VMHC | **0.002^*^** | **0.029^*^** | **0.040^*^** | 0.214 | 0.916 | 0.063 | 0.153 | 0.304 | 0.119 | 0.529 | 0.348 | 0.244 | 0.405 | 0.289 | 0.940 |
| ALFF+DC+ReHo | **0.005^*^** | 0.054 | 0.078 | 0.361 | 0.908 | 0.125 | 0.252 | 0.533 | 0.221 | 0.889 | 0.611 | 0.421 | 0.707 | 0.502 | 0.577 |
| ALFF+DC+VMHC | **0.009^*^** | 0.077 | 0.115 | 0.489 | 0.904 | 0.182 | 0.339 | 0.723 | 0.312 | 0.841 | 0.825 | 0.572 | 0.944 | 0.680 | 0.326 |
| ALFF+ReHo+VMHC | **0.002^*^** | **0.025^*^** | **0.034^*^** | 0.185 | 0.917 | 0.053 | 0.134 | 0.258 | 0.101 | 0.443 | 0.294 | 0.210 | 0.341 | 0.247 | 0.918 |
| DC+ReHo+VMHC | **0.011^*^** | 0.087 | 0.131 | 0.527 | 0.901 | 0.206 | 0.367 | 0.770 | 0.345 | 0.806 | 0.872 | 0.614 | 0.989 | 0.726 | 0.340 |
| ALFF+DC+ReHo+VMHC | **0.008^*^** | 0.070 | 0.103 | 0.441 | 0.904 | 0.164 | 0.307 | 0.647 | 0.281 | 0.965 | 0.737 | 0.514 | 0.843 | 0.610 | 0.473 |

Note: * , P ＜0.05; **, P＜0.001; ALFF, Amplitude of Low Frequency Fluctuations; ReHo, Regional Homogeneity; DC, Degree centrality; VMHC, Voxel-Mirrored Homotopic Connectivity.

Figure S2. The SHAP summary plot of the Logistic Regression (LR) models.


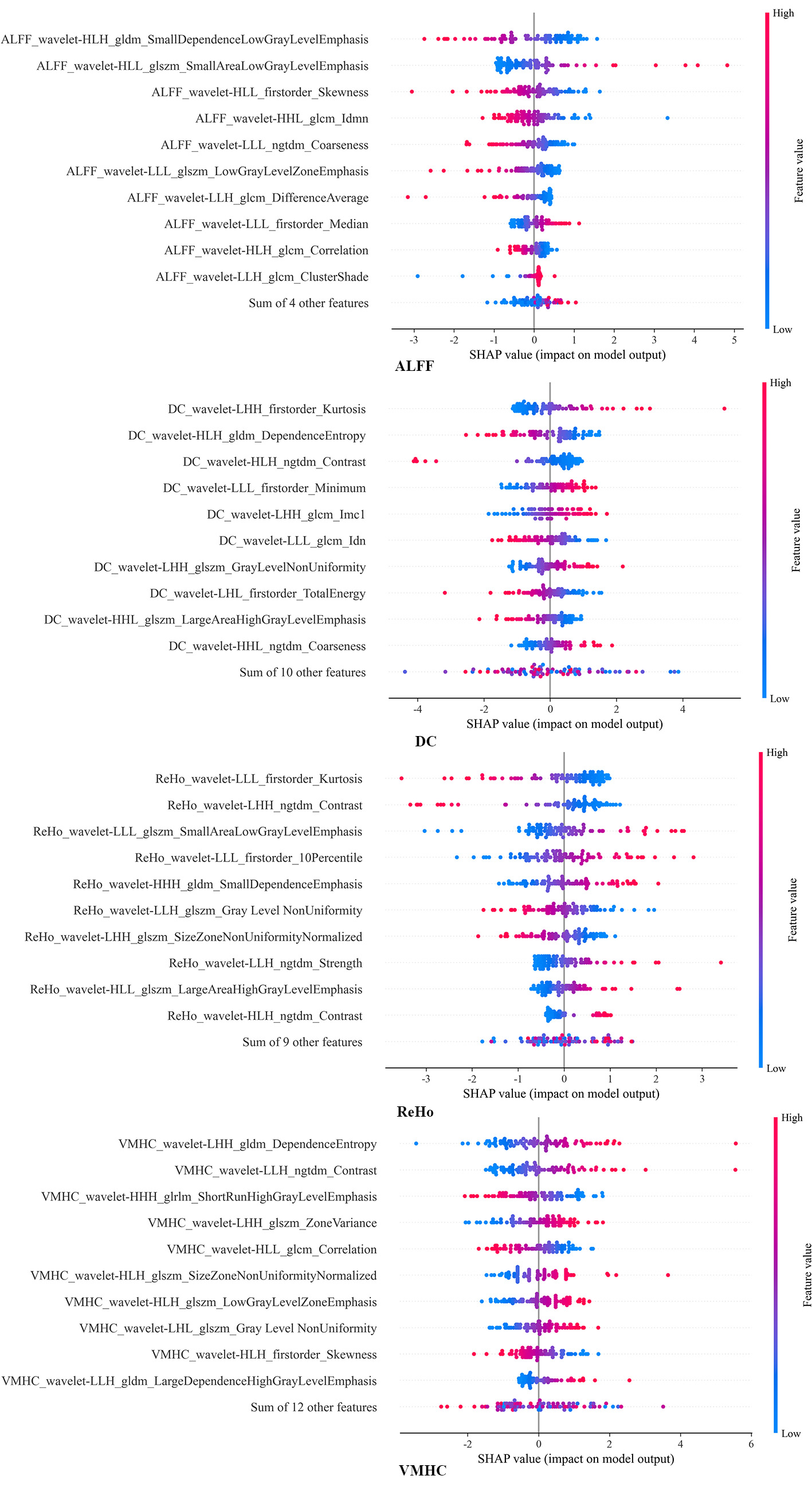


Figure S2. The SHAP summary plot of the Logistic Regression (LR) models constructed by single imaging index.

Note: ALFF, Amplitude of Low Frequency Fluctuations; ReHo, Regional Homogeneity; DC, Degree centrality; VMHC, Voxel-Mirrored Homotopic Connectivity; SHAP, Shapley's additive explanations.


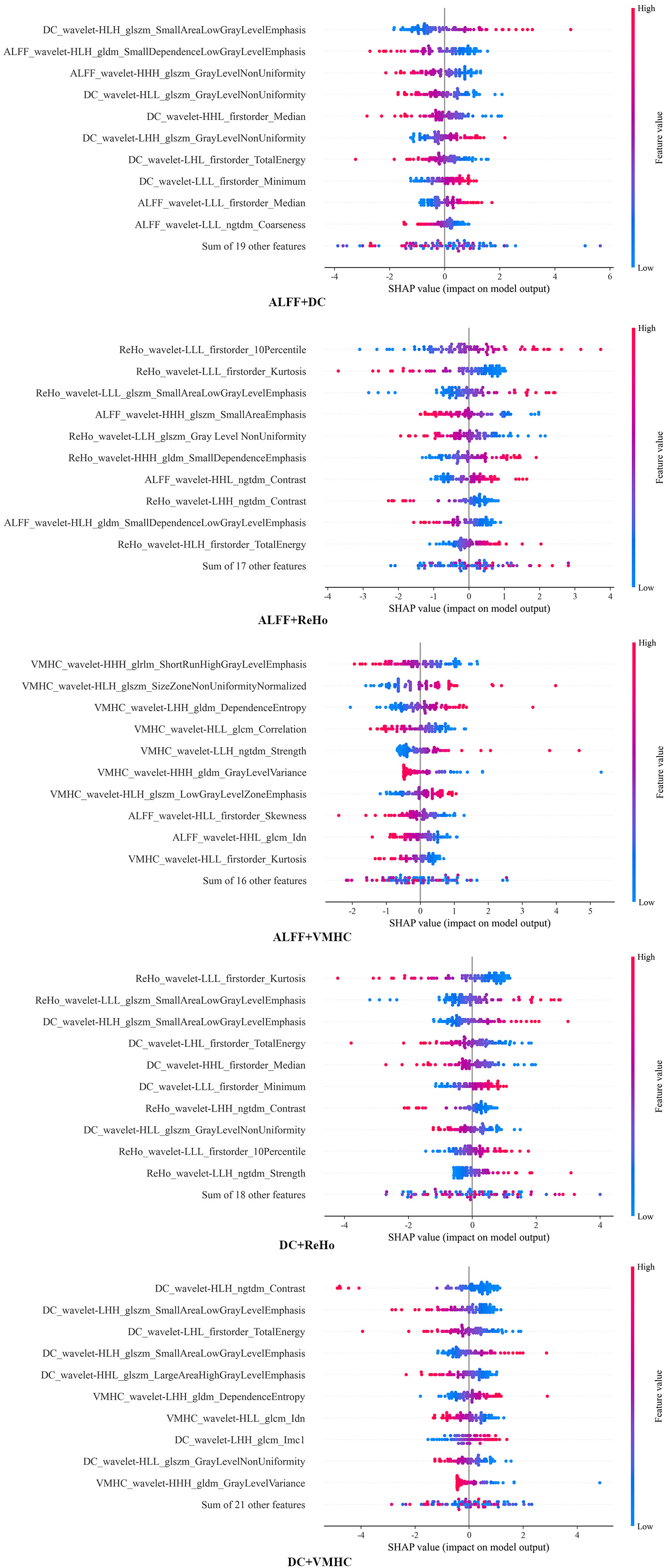


Figure S3. The SHAP summary plot of the Logistic Regression (LR) models constructed by two imaging indicators.

Note: ALFF, Amplitude of Low Frequency Fluctuations; ReHo, Regional Homogeneity; DC, Degree centrality; VMHC, Voxel-Mirrored Homotopic Connectivity; SHAP, Shapley's additive explanations.


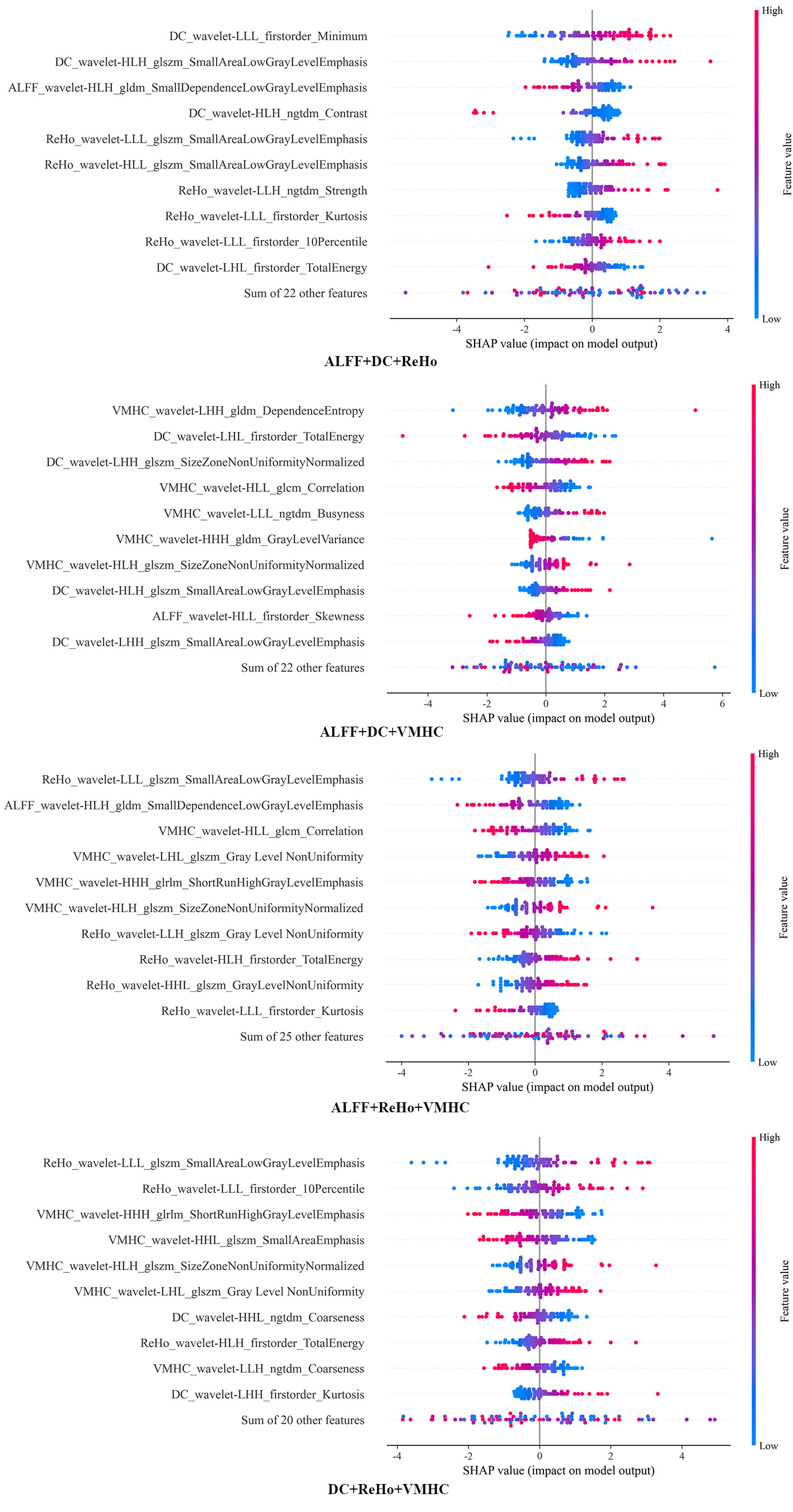


Figure S4. The SHAP summary plot of the Logistic Regression (LR) models constructed by three kinds of imaging index.

Note: ALFF, Amplitude of Low Frequency Fluctuations; ReHo, Regional Homogeneity; DC, Degree centrality; VMHC, Voxel-Mirrored Homotopic Connectivity; SHAP, Shapley's additive explanations.

Table S22. The Results of partial correlation analysis in PD-CI group.

|  |  | PDQ-39 | HAMA | HAMD | MOCA | NMSS | UPDRS-III |
| --- | --- | --- | --- | --- | --- | --- | --- |
| ALFF_wavelet_HHH_glszm_GrayLevelNon  _Uniformity | r | 0.028 | -0.082 | -0.034 | 0.148 | 0.097 | -0.068 |
|  | p | 0.847 | 0.568 | 0.813 | 0.300 | 0.497 | 0.634 |
| ALFF_wavelet-HHL_firstorder_Kurtosis | r | 0.187 | -0.233 | -0.200 | 0.185 | 0.030 | 0.053 |
|  | p | 0.188 | 0.099 | 0.159 | 0.193 | 0.835 | 0.713 |
| ALFF_wavelet-HHL_glcm_Idmn | r | -0.034 | -0.300 | -0.254 | 0.376 | -0.173 | 0.055 |
|  | p | 0.810 | 0.032 | 0.072 | 0.007 | 0.225 | 0.703 |
| ALFF_wavelet-HHL_glcm_Idn | r | 0.042 | -0.377 | -0.337 | 0.211 | -0.165 | 0.117 |
|  | p | 0.770 | 0.006 | 0.015 | 0.138 | 0.248 | 0.413 |
| ALFF_wavelet-HLH_glcm_Correlation | r | -0.089 | -0.249 | -0.203 | 0.206 | -0.241 | 0.058 |
|  | p | 0.533 | 0.078 | 0.154 | 0.147 | 0.089 | 0.683 |
| ALFF_wavelet-HLH_gldm_Small  _Dependence Low Gray Level Emphasis | r | 0.048 | 0.180 | 0.083 | -0.116 | -0.011 | -0.173 |
|  | p | 0.736 | 0.206 | 0.561 | 0.418 | 0.940 | 0.224 |
| ALFF_wavelet-HLL_firstorder_Skewness | r | -0.024 | -0.118 | -0.068 | 0.265 | 0.018 | 0.038 |
|  | p | 0.865 | 0.409 | 0.633 | 0.060 | 0.899 | 0.793 |
| ALFF_wavelet-LLL_ngtdm_Coarseness | r | -0.020 | 0.055 | 0.035 | -0.306 | -0.012 | 0.016 |
|  | p | 0.890 | 0.700 | 0.806 | 0.029 | 0.932 | 0.911 |
| DC_wavelet-HHL_glszm_Large Area High  _Gray Level Emphasis | r | 0.055 | 0.273 | 0.169 | -0.001 | 0.165 | 0.046 |
|  | p | 0.703 | 0.052 | 0.236 | 0.995 | 0.248 | 0.751 |
| DC_wavelet-HLH_firstorder_Skewness | r | 0.065 | -0.103 | 0.058 | 0.019 | -0.154 | -0.063 |
|  | p | 0.650 | 0.473 | 0.687 | 0.892 | 0.282 | 0.660 |
| DC_wavelet-HLH_gldm_Dependence  _Entropy | r | 0.052 | -0.120 | -0.088 | 0.159 | 0.026 | -0.075 |
|  | p | 0.717 | 0.402 | 0.541 | 0.264 | 0.857 | 0.602 |
| DC_wavelet-HLH_glszm_Small Area Low  _Gray Level Emphasis | r | -0.233 | -0.107 | -0.087 | -0.316 | -0.034 | -0.108 |
|  | p | 0.099 | 0.454 | 0.544 | 0.024 | 0.811 | 0.449 |
| DC_wavelet-LHH_firstorder_Kurtosis | r | -0.071 | 0.347 | 0.220 | -0.220 | -0.036 | -0.186 |
|  | p | 0.622 | 0.013 | 0.121 | 0.121 | 0.803 | 0.192 |
| DC_wavelet-LHH_glcm_Imc1 | r | 0.083 | -0.022 | -0.030 | 0.165 | 0.013 | 0.007 |
|  | p | 0.563 | 0.876 | 0.836 | 0.247 | 0.928 | 0.964 |
| DC_wavelet-LHH_glszm_Small Area  _Low Gray Level Emphasis | r | -0.062 | -0.107 | 0.011 | -0.084 | 0.114 | -0.039 |
|  | p | 0.665 | 0.455 | 0.939 | 0.559 | 0.428 | 0.788 |
| DC_wavelet-LHL_firstorder_TotalEnergy | r | 0.289 | 0.293 | 0.335 | -0.076 | 0.410 | 0.134 |
|  | p | 0.040 | 0.037 | 0.016 | 0.595 | 0.003 | 0.347 |
| ReHo_wavelet-HLH_firstorder_TotalEnergy | r | 0.271 | 0.082 | 0.057 | -0.062 | 0.186 | 0.021 |
|  | p | 0.054 | 0.566 | 0.692 | 0.664 | 0.190 | 0.885 |
| ReHo_wavelet-HLL_glszm_Large Area High  _Gray Level Emphasis | r | -0.043 | -0.037 | 0.022 | -0.020 | -0.086 | -0.015 |
|  | p | 0.762 | 0.799 | 0.881 | 0.891 | 0.550 | 0.916 |
| ReHo_wavelet-LLH_glszm_Gray Level  _NonUniformity | r | -0.034 | 0.078 | 0.055 | 0.044 | 0.177 | 0.174 |
|  | p | 0.815 | 0.585 | 0.701 | 0.761 | 0.215 | 0.221 |
| ReHo_wavelet-LLL_firstorder_10Percentile | r | -0.012 | 0.050 | 0.019 | 0.266 | -0.048 | -0.023 |
|  | p | 0.932 | 0.730 | 0.896 | 0.059 | 0.740 | 0.875 |
| ReHo_wavelet-LLL_firstorder_Minimum | r | 0.039 | -0.067 | -0.029 | 0.275 | -0.082 | -0.131 |
|  | p | 0.787 | 0.638 | 0.839 | 0.051 | 0.568 | 0.360 |
| ReHo_wavelet-LLL_glszm_Small Area Low  _Gray Level Emphasis | r | -0.031 | -0.045 | -0.080 | 0.074 | -0.022 | -0.050 |
|  | p | 0.831 | 0.754 | 0.575 | 0.604 | 0.877 | 0.726 |
| VMHC_wavelet-HHH_gldm_Gray Level  Variance | r | -0.006 | 0.054 | 0.005 | -0.115 | 0.011 | -0.265 |
|  | p | 0.968 | 0.709 | 0.973 | 0.422 | 0.941 | 0.060 |
| VMHC_wavelet-HHH_glrlm_Short Run  _High Gray Level Emphasis | r | 0.321 | 0.293 | 0.212 | 0.104 | 0.256 | 0.322 |
|  | p | 0.022 | 0.037 | 0.136 | 0.467 | 0.070 | 0.021 |
| VMHC_wavelet-HLH_glcm_ClusterShade | r | -0.267 | -0.048 | -0.091 | 0.042 | -0.091 | -0.199 |
|  | p | 0.058 | 0.739 | 0.523 | 0.770 | 0.525 | 0.161 |
| VMHC_wavelet-HLH_glszm_Size Zone  _NonUniformity Normalized | r | 0.063 | -0.012 | 0.083 | -0.131 | 0.142 | 0.155 |
|  | p | 0.660 | 0.932 | 0.561 | 0.359 | 0.322 | 0.276 |
| VMHC_wavelet-HLL_glcm_Correlation | r | -0.145 | -0.150 | -0.292 | 0.155 | -0.277 | -0.004 |
|  | p | 0.311 | 0.295 | 0.038 | 0.277 | 0.049 | 0.979 |
| VMHC_wavelet-LHH_gldm_Dependence  _Entropy | r | 0.091 | -0.110 | -0.105 | 0.149 | 0.044 | 0.052 |
|  | p | 0.523 | 0.442 | 0.464 | 0.297 | 0.758 | 0.716 |
| VMHC_wavelet-LHH_glszm_Small Area  _Low Gray Level Emphasis | r | -0.046 | -0.227 | -0.185 | 0.321 | 0.087 | -0.033 |
|  | p | 0.750 | 0.109 | 0.194 | 0.022 | 0.544 | 0.819 |
| VMHC_wavelet-LHL_glszm_Gray Level  _NonUniformity | r | 0.057 | 0.195 | 0.166 | -0.008 | 0.118 | -0.042 |
|  | p | 0.691 | 0.170 | 0.245 | 0.955 | 0.411 | 0.772 |
| VMHC_wavelet-LLL_firstorder_Total  _Energy | r | -0.115 | -0.143 | -0.056 | -0.120 | -0.102 | -0.035 |
|  | p | 0.422 | 0.318 | 0.698 | 0.401 | 0.478 | 0.805 |
| VMHC_ wavelet-LLL_glcm_Correlation | r | -0.093 | -0.101 | -0.085 | -0.217 | -0.182 | 0.080 |
|  | p | 0.518 | 0.481 | 0.554 | 0.126 | 0.202 | 0.578 |

Note: glrlm, gray-level run-length matrix, glszm, gray-level size zone matrix, ngtdm, neighborhood gray-tone difference matrix; gldm, gray-level dependence matrix; ALFF, Amplitude of Low Frequency Fluctuations; ReHo, Regional Homogeneity; DC, Degree centrality; VMHC, Voxel-Mirrored Homotopic Connectivity; HAMD, Hamilton Depression Scale; HAMA, Hamilton Anxiety Scale; NMSS, Non-Motor Symptom Scale; UPDRS III, Unified Parkinson's Disease Rating Scale Part III; PDQ-39, Parkinson's disease questionnaire-39; MoCA, Montreal Cognitive Assessment;

Table S23. The Results of partial correlation analysis in PD-CP group.

|  |  | PDQ-39 | HAMA | HAMD | MOCA | NMSS | UPDRS-III |
| --- | --- | --- | --- | --- | --- | --- | --- |
| ALFF_wavelet_HHH_glszm_GrayLevelNon | r | 0.198 | 0.105 | 0.246 | -0.306 | 0.075 | 0.073 |
| _Uniformity | p | 0.276 | 0.568 | 0.175 | 0.088 | 0.683 | 0.691 |
| ALFF_wavelet-HHL_firstorder_Kurtosis | r | 0.257 | 0.165 | 0.061 | -0.080 | 0.301 | 0.297 |
|  | p | 0.156 | 0.367 | 0.741 | 0.664 | 0.094 | 0.099 |
| ALFF_wavelet-HHL_glcm_Idmn | r | 0.250 | 0.138 | 0.078 | 0.131 | 0.244 | 0.277 |
|  | p | 0.168 | 0.453 | 0.670 | 0.473 | 0.178 | 0.124 |
| ALFF_wavelet-HHL_glcm_Idn | r | 0.054 | -0.014 | -0.102 | 0.212 | 0.075 | 0.148 |
|  | p | 0.768 | 0.940 | 0.577 | 0.244 | 0.681 | 0.418 |
| ALFF_wavelet-HLH_glcm_Correlation | r | -0.215 | -0.182 | -0.352 | 0.133 | -0.147 | -0.227 |
|  | p | 0.236 | 0.318 | 0.048 | 0.467 | 0.421 | 0.211 |
| ALFF_wavelet-HLH_gldm_Small | r | -0.211 | -0.154 | 0.030 | 0.187 | -0.174 | 0.091 |
| _Dependence Low Gray Level Emphasis | p | 0.246 | 0.400 | 0.868 | 0.305 | 0.341 | 0.620 |
| ALFF_wavelet-HLL_firstorder_Skewness | r | 0.133 | -0.010 | -0.059 | -0.043 | -0.078 | -0.008 |
|  | p | 0.468 | 0.957 | 0.749 | 0.816 | 0.673 | 0.966 |
| ALFF_wavelet-LLL_ngtdm_Coarseness | r | -0.066 | -0.192 | -0.176 | 0.138 | -0.217 | -0.220 |
|  | p | 0.720 | 0.293 | 0.336 | 0.450 | 0.233 | 0.226 |
| DC_wavelet-HHL_glszm_Large Area High | r | 0.110 | 0.064 | 0.009 | -0.151 | 0.152 | 0.155 |
| _Gray Level Emphasis | p | 0.550 | 0.728 | 0.960 | 0.409 | 0.405 | 0.398 |
| DC_wavelet-HLH_firstorder_Skewness | r | -0.300 | -0.421 | -0.557 | -0.015 | -0.415 | -0.264 |
|  | p | 0.096 | 0.016 | 0.001 | 0.937 | 0.018 | 0.145 |
| DC_wavelet-HLH_gldm_Dependence | r | 0.185 | 0.156 | -0.028 | -0.037 | 0.209 | 0.104 |
| _Entropy | p | 0.309 | 0.395 | 0.878 | 0.839 | 0.250 | 0.570 |
| DC_wavelet-HLH_glszm_Small Area Low | r | -0.247 | -0.233 | 0.019 | -0.066 | -0.381 | -0.423 |
| _Gray Level Emphasis | p | 0.173 | 0.200 | 0.917 | 0.718 | 0.032 | 0.016 |
| DC_wavelet-LHH_firstorder_Kurtosis | r | -0.029 | -0.041 | 0.207 | 0.040 | -0.200 | -0.064 |
|  | p | 0.876 | 0.823 | 0.255 | 0.826 | 0.273 | 0.726 |
| DC_wavelet-LHH_glcm_Imc1 | r | -0.100 | -0.250 | -0.041 | 0.242 | 0.077 | 0.060 |
|  | p | 0.587 | 0.168 | 0.824 | 0.183 | 0.675 | 0.745 |
| DC_wavelet-LHH_glszm_Small Area | r | -0.110 | -0.371 | -0.235 | 0.029 | -0.119 | 0.004 |
| _Low Gray Level Emphasis | p | 0.550 | 0.037 | 0.196 | 0.875 | 0.515 | 0.984 |
| DC_wavelet-LHL_firstorder_TotalEnergy | r | 0.573 | 0.256 | 0.086 | -0.172 | 0.281 | 0.483 |
|  | p | 0.001 | 0.158 | 0.638 | 0.346 | 0.119 | 0.005 |
| ReHo_wavelet-HLH_firstorder_TotalEnergy | r | 0.287 | 0.266 | 0.472 | 0.121 | 0.301 | 0.182 |
|  | p | 0.112 | 0.142 | 0.006 | 0.509 | 0.094 | 0.320 |
| ReHo_wavelet-HLL_glszm_Large Area High | r | 0.099 | -0.020 | -0.115 | -0.126 | -0.132 | -0.232 |
| _Gray Level Emphasis | p | 0.591 | 0.912 | 0.532 | 0.491 | 0.471 | 0.202 |
| ReHo_wavelet-LLH_glszm_Gray Level | r | -0.097 | -0.302 | -0.047 | 0.084 | -0.157 | 0.129 |
| _NonUniformity | p | 0.596 | 0.093 | 0.800 | 0.648 | 0.390 | 0.483 |
| ReHo_wavelet-LLL_firstorder_10Percentile | r | 0.107 | -0.080 | 0.075 | 0.090 | -0.123 | 0.083 |
|  | p | 0.561 | 0.663 | 0.683 | 0.623 | 0.502 | 0.652 |
| ReHo_wavelet-LLL_firstorder_Minimum | r | -0.012 | -0.024 | 0.089 | 0.029 | -0.271 | -0.117 |
|  | p | 0.947 | 0.896 | 0.628 | 0.873 | 0.133 | 0.523 |
| ReHo_wavelet-LLL_glszm_Small Area Low | r | -0.212 | -0.150 | 0.034 | -0.037 | -0.295 | -0.332 |
| _Gray Level Emphasis | p | 0.244 | 0.412 | 0.854 | 0.840 | 0.101 | 0.063 |
| VMHC_wavelet-HHH_gldm_Gray Level | r | 0.026 | 0.013 | 0.135 | -0.143 | 0.029 | -0.232 |
| Variance | p | 0.886 | 0.942 | 0.460 | 0.433 | 0.874 | 0.201 |
| VMHC_wavelet-HHH_glrlm_Short Run | r | 0.409 | 0.241 | 0.282 | -0.090 | 0.081 | 0.124 |
| _High Gray Level Emphasis | p | 0.020 | 0.184 | 0.117 | 0.625 | 0.657 | 0.498 |
| VMHC_wavelet-HLH_glcm_ClusterShade | r | 0.038 | 0.134 | 0.080 | -0.086 | 0.181 | 0.135 |
|  | p | 0.836 | 0.465 | 0.662 | 0.641 | 0.321 | 0.461 |
| VMHC_wavelet-HLH_glszm_Size Zone | r | -0.150 | 0.031 | -0.065 | -0.054 | -0.118 | -0.335 |
| _NonUniformity Normalized | p | 0.414 | 0.866 | 0.725 | 0.769 | 0.518 | 0.061 |
| VMHC_wavelet-HLL_glcm_Correlation | r | -0.335 | -0.244 | -0.207 | 0.147 | -0.181 | -0.195 |
|  | p | 0.061 | 0.179 | 0.256 | 0.423 | 0.322 | 0.286 |
| VMHC_wavelet-LHH_gldm_Dependence | r | 0.000 | -0.214 | -0.057 | 0.166 | -0.173 | -0.208 |
| _Entropy | p | 1.000 | 0.240 | 0.758 | 0.363 | 0.344 | 0.254 |
| VMHC_wavelet-LHH_glszm_Small Area | r | -0.106 | -0.092 | 0.089 | 0.212 | 0.032 | 0.160 |
| _Low Gray Level Emphasis | p | 0.562 | 0.618 | 0.628 | 0.244 | 0.863 | 0.383 |
| VMHC_wavelet-LHL_glszm_Gray Level | r | -0.027 | 0.020 | -0.159 | -0.141 | 0.090 | 0.204 |
| _NonUniformity | p | 0.885 | 0.914 | 0.383 | 0.440 | 0.624 | 0.263 |
| VMHC_wavelet-LLL_firstorder_Total | r | -0.177 | -0.197 | -0.008 | 0.121 | 0.042 | -0.021 |
| _Energy | p | 0.332 | 0.279 | 0.964 | 0.511 | 0.820 | 0.907 |
| VMHC_ wavelet-LLL_glcm_Correlation | r | 0.087 | 0.012 | 0.027 | 0.157 | -0.043 | -0.140 |
|  | p | 0.635 | 0.950 | 0.883 | 0.390 | 0.816 | 0.446 |

Note: glrlm, gray-level run-length matrix, glszm, gray-level size zone matrix, ngtdm, neighborhood gray-tone difference matrix; gldm, gray-level dependence matrix; ALFF, Amplitude of Low Frequency Fluctuations; ReHo, Regional Homogeneity; DC, Degree centrality; VMHC, Voxel-Mirrored Homotopic Connectivity; HAMD, Hamilton Depression Scale; HAMA, Hamilton Anxiety Scale; NMSS, Non-Motor Symptom Scale; UPDRS III, Unified Parkinson's Disease Rating Scale Part III; PDQ-39, Parkinson's disease questionnaire-39; MoCA, Montreal Cognitive Assessment;

Table S24. The Results of partial correlation analysis in all PD patients.

|  |  | PDQ-39 | HAMA | HAMD | MOCA | NMSS | UPDRS-III |
| --- | --- | --- | --- | --- | --- | --- | --- |
| ALFF_wavelet_HHH_glszm_GrayLevelNon_Uniformity | r | 0.193 | 0.143 | 0.053 | 0.070 | 0.087 | -0.029 |
|  | p | 0.077 | 0.192 | 0.632 | 0.527 | 0.429 | 0.794 |
| ALFF_wavelet-HHL_firstorder_Kurtosis | r | -0.070 | -0.020 | -0.030 | 0.000 | 0.012 | -0.072 |
|  | p | 0.526 | 0.856 | 0.785 | 0.997 | 0.915 | 0.512 |
| ALFF_wavelet-HHL_glcm_Idmn | r | -0.171 | -0.211 | -0.237 | 0.143 | -0.123 | -0.042 |
|  | p | 0.118 | 0.052 | 0.029 | 0.192 | 0.261 | 0.704 |
| ALFF_wavelet-HHL_glcm_Idn | r | -0.126 | -0.096 | -0.116 | -0.167 | -0.044 | 0.066 |
|  | p | 0.250 | 0.383 | 0.290 | 0.127 | 0.689 | 0.551 |
| ALFF_wavelet-HLH_glcm_Correlation | r | -0.095 | -0.153 | -0.151 | -0.400 | -0.036 | -0.025 |
|  | p | 0.387 | 0.163 | 0.167 | 0.000 | 0.747 | 0.819 |
| ALFF_wavelet-HLH_gldm_Small  _Dependence Low Gray Level Emphasis | r | 0.092 | 0.129 | 0.028 | 0.172 | -0.040 | -0.046 |
|  | p | 0.403 | 0.239 | 0.798 | 0.116 | 0.716 | 0.674 |
| ALFF_wavelet-HLL_firstorder_Skewness | r | -0.101 | -0.197 | -0.218 | 0.138 | -0.103 | -0.003 |
|  | p | 0.356 | 0.071 | 0.045 | 0.206 | 0.349 | 0.981 |
| ALFF_wavelet-LLL_ngtdm_Coarseness | r | 0.015 | 0.042 | 0.065 | -0.240 | -0.058 | 0.118 |
|  | p | 0.889 | 0.704 | 0.554 | 0.027 | 0.597 | 0.284 |
| DC_wavelet-HHL_glszm_Large Area High  _Gray Level Emphasis | r | -0.024 | -0.026 | 0.067 | 0.214 | 0.034 | 0.006 |
|  | p | 0.826 | 0.815 | 0.542 | 0.049 | 0.756 | 0.959 |
| DC_wavelet-HLH_firstorder_Skewness | r | 0.094 | 0.035 | 0.114 | -0.268 | 0.117 | 0.031 |
|  | p | 0.393 | 0.750 | 0.299 | 0.013 | 0.285 | 0.775 |
| DC_wavelet-HLH_gldm_Dependence  _Entropy | r | -0.044 | -0.124 | -0.028 | 0.009 | -0.071 | -0.118 |
|  | p | 0.688 | 0.259 | 0.798 | 0.933 | 0.519 | 0.283 |
| DC_wavelet-HLH_glszm_Small Area Low  _Gray Level Emphasis | r | 0.109 | -0.006 | -0.026 | -0.168 | 0.113 | 0.152 |
|  | p | 0.323 | 0.960 | 0.815 | 0.124 | 0.301 | 0.164 |
| DC_wavelet-LHH_firstorder_Kurtosis | r | 0.230 | 0.234 | 0.228 | 0.146 | 0.163 | 0.096 |
|  | p | 0.034 | 0.031 | 0.036 | 0.182 | 0.135 | 0.382 |
| DC_wavelet-LHH_glcm_Imc1 | r | -0.181 | -0.064 | -0.067 | 0.183 | -0.314 | -0.193 |
|  | p | 0.098 | 0.563 | 0.542 | 0.093 | 0.003 | 0.077 |
| DC_wavelet-LHH_glszm_Small Area | r | -0.144 | 0.048 | 0.049 | 0.011 | -0.190 | -0.142 |
| _Low Gray Level Emphasis | p | 0.189 | 0.661 | 0.656 | 0.922 | 0.082 | 0.194 |
| DC_wavelet-LHL_firstorder_TotalEnergy | r | -0.049 | 0.039 | -0.006 | 0.432 | -0.048 | -0.168 |
|  | p | 0.653 | 0.721 | 0.958 | 0.000 | 0.663 | 0.125 |
| ReHo_wavelet-HLH_firstorder_TotalEnergy | r | -0.069 | -0.090 | -0.096 | -0.072 | -0.087 | -0.109 |
|  | p | 0.527 | 0.411 | 0.384 | 0.515 | 0.429 | 0.323 |
| ReHo_wavelet-HLL_glszm_Large Area High  _Gray Level Emphasis | r | 0.021 | -0.080 | -0.083 | -0.201 | 0.025 | 0.062 |
|  | p | 0.851 | 0.466 | 0.449 | 0.065 | 0.822 | 0.572 |
| ReHo_wavelet-LLH_glszm_Gray Level  _NonUniformity | r | 0.038 | -0.025 | 0.042 | 0.203 | -0.143 | -0.023 |
|  | p | 0.730 | 0.822 | 0.704 | 0.063 | 0.192 | 0.831 |
| ReHo_wavelet-LLL_firstorder_10Percentile | r | -0.048 | -0.287 | -0.277 | -0.043 | -0.068 | -0.015 |
|  | p | 0.662 | 0.008 | 0.010 | 0.694 | 0.536 | 0.895 |
| ReHo_wavelet-LLL_firstorder_Minimum | r | 0.004 | -0.206 | -0.194 | -0.114 | -0.065 | 0.031 |
|  | p | 0.971 | 0.058 | 0.075 | 0.299 | 0.552 | 0.780 |
| ReHo_wavelet-LLL_glszm_Small Area Low  _Gray Level Emphasis | r | -0.079 | 0.001 | 0.009 | -0.122 | -0.090 | -0.129 |
|  | p | 0.474 | 0.995 | 0.935 | 0.267 | 0.411 | 0.240 |
| VMHC_wavelet-HHH_gldm_Gray Level  Variance | r | -0.013 | 0.054 | 0.054 | 0.104 | -0.186 | -0.306 |
|  | p | 0.903 | 0.626 | 0.621 | 0.346 | 0.089 | 0.004 |
| VMHC_wavelet-HHH_glrlm_Short Run  _High Gray Level Emphasis | r | -0.226 | -0.064 | -0.162 | 0.239 | -0.092 | -0.106 |
|  | p | 0.038 | 0.562 | 0.138 | 0.028 | 0.402 | 0.336 |
| VMHC_wavelet-HLH_glcm_ClusterShade | r | -0.138 | -0.064 | -0.114 | -0.030 | -0.002 | -0.125 |
|  | p | 0.208 | 0.561 | 0.299 | 0.785 | 0.989 | 0.256 |
| VMHC_wavelet-HLH_glszm_Size Zone  _NonUniformity Normalized | r | 0.126 | 0.009 | -0.051 | -0.060 | -0.029 | 0.197 |
|  | p | 0.250 | 0.938 | 0.645 | 0.586 | 0.790 | 0.070 |
| VMHC_wavelet-HLL_glcm_Correlation | r | -0.066 | -0.133 | -0.013 | 0.128 | 0.008 | -0.001 |
|  | p | 0.549 | 0.226 | 0.908 | 0.242 | 0.942 | 0.991 |
| VMHC_wavelet-LHH_gldm_Dependence  _Entropy | r | 0.034 | -0.020 | -0.038 | -0.012 | -0.078 | -0.166 |
|  | p | 0.756 | 0.856 | 0.729 | 0.915 | 0.477 | 0.130 |
| VMHC_wavelet-LHH_glszm_Small Area  _Low Gray Level Emphasis | r | -0.128 | -0.177 | -0.221 | 0.179 | -0.138 | 0.020 |
|  | p | 0.242 | 0.104 | 0.043 | 0.101 | 0.209 | 0.856 |
| VMHC_wavelet-LHL_glszm_Gray Level  _NonUniformity | r | 0.264 | 0.061 | 0.149 | 0.257 | 0.141 | 0.158 |
|  | p | 0.015 | 0.582 | 0.175 | 0.017 | 0.198 | 0.150 |
| VMHC_wavelet-LLL_firstorder_Total  _Energy | r | -0.061 | -0.008 | -0.047 | -0.244 | -0.096 | -0.106 |
|  | p | 0.577 | 0.945 | 0.667 | 0.024 | 0.380 | 0.333 |
| VMHC_ wavelet-LLL_glcm_Correlation | r | -0.089 | -0.003 | 0.029 | -0.103 | 0.035 | 0.104 |
|  | p | 0.420 | 0.981 | 0.790 | 0.347 | 0.749 | 0.342 |

Note: glrlm, gray-level run-length matrix, glszm, gray-level size zone matrix, ngtdm, neighborhood gray-tone difference matrix; gldm, gray-level dependence matrix; ALFF, Amplitude of Low Frequency Fluctuations; ReHo, Regional Homogeneity; DC, Degree centrality; VMHC, Voxel-Mirrored Homotopic Connectivity; HAMD, Hamilton Depression Scale; HAMA, Hamilton Anxiety Scale; NMSS, Non-Motor Symptom Scale; UPDRS III, Unified Parkinson's Disease Rating Scale Part III; PDQ-39, Parkinson's disease questionnaire-39; MoCA, Montreal Cognitive Assessment;
